# Proximity Ligation Assays for *In Situ* Detection of Innate Immune Activation: Focus on *In Vitro-*Transcribed mRNA

**DOI:** 10.1016/j.omtn.2018.11.002

**Published:** 2018-11-20

**Authors:** Emmeline L. Blanchard, Kristin H. Loomis, Sushma M. Bhosle, Daryll Vanover, Patrick Baumhof, Bruno Pitard, Chiara Zurla, Philip J. Santangelo

**Affiliations:** 1 Wallace H. Coulter Department of Biomedical Engineering, Georgia Institute of Technology and Emory University, 313 Ferst Drive, UA Whitaker Building, Atlanta, GA 30332, USA; 2 CureVac, Paul-Ehrlich-Straße 15, 72076 Tübingen, Germany; 3 In-Cell-Art, 21 rue de la Noue Bras de Fer, 44200 Nantes, France

**Keywords:** *in vitro*-transcribed mRNA, innate immune responses, proximity ligation assay, protein-protein interactions

## Abstract

The characterization of innate immune activation is crucial for vaccine and therapeutic development, including RNA-based vaccines, a promising approach. Current measurement methods quantify type I interferon and inflammatory cytokine production, but they do not allow for the isolation of individual pathways, do not provide kinetic activation or spatial information within tissues, and cannot be translated into clinical studies. Here we demonstrated the use of proximity ligation assays (PLAs) to detect pattern recognition receptor (PRR) activation in cells and in tissue samples. First, we validated PLA’s sensitivity and specificity using well-characterized soluble agonists. Next, we characterized PRR activation from *in vitro*-transcribed (IVT) mRNAs, as well as the effect of sequence and base modifications *in vitro*. Finally, we established the measurement of PRR activation in tissue sections via PLA upon IVT mRNA intramuscular (i.m.) injection in mice. Overall, our results indicate that PLA is a valuable, versatile, and sensitive tool to monitor PRR activation for vaccine, adjuvant, and therapeutic screening.

## Graphical Abstract

**Figure:**
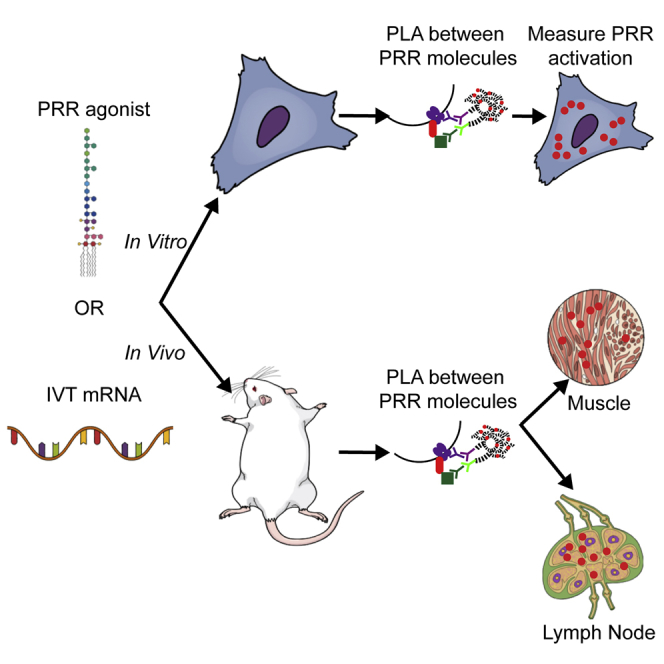

## Introduction

The innate immune system is the cell’s frontline mechanism of protection against foreign pathogens.^1^, ^2^, ^3^, ^4^, ^5^ In the context of vaccine development, innate immune response activation is desirable to develop protective immunity.^6^, ^7^, ^8^ Conversely, for gene therapy, its activation must be avoided.^9^, ^10^, ^11^, ^12^ Thus, characterizing the activation of the innate immune response is fundamental to understanding the efficacy of vaccines and therapeutics.

**Figure 1:**
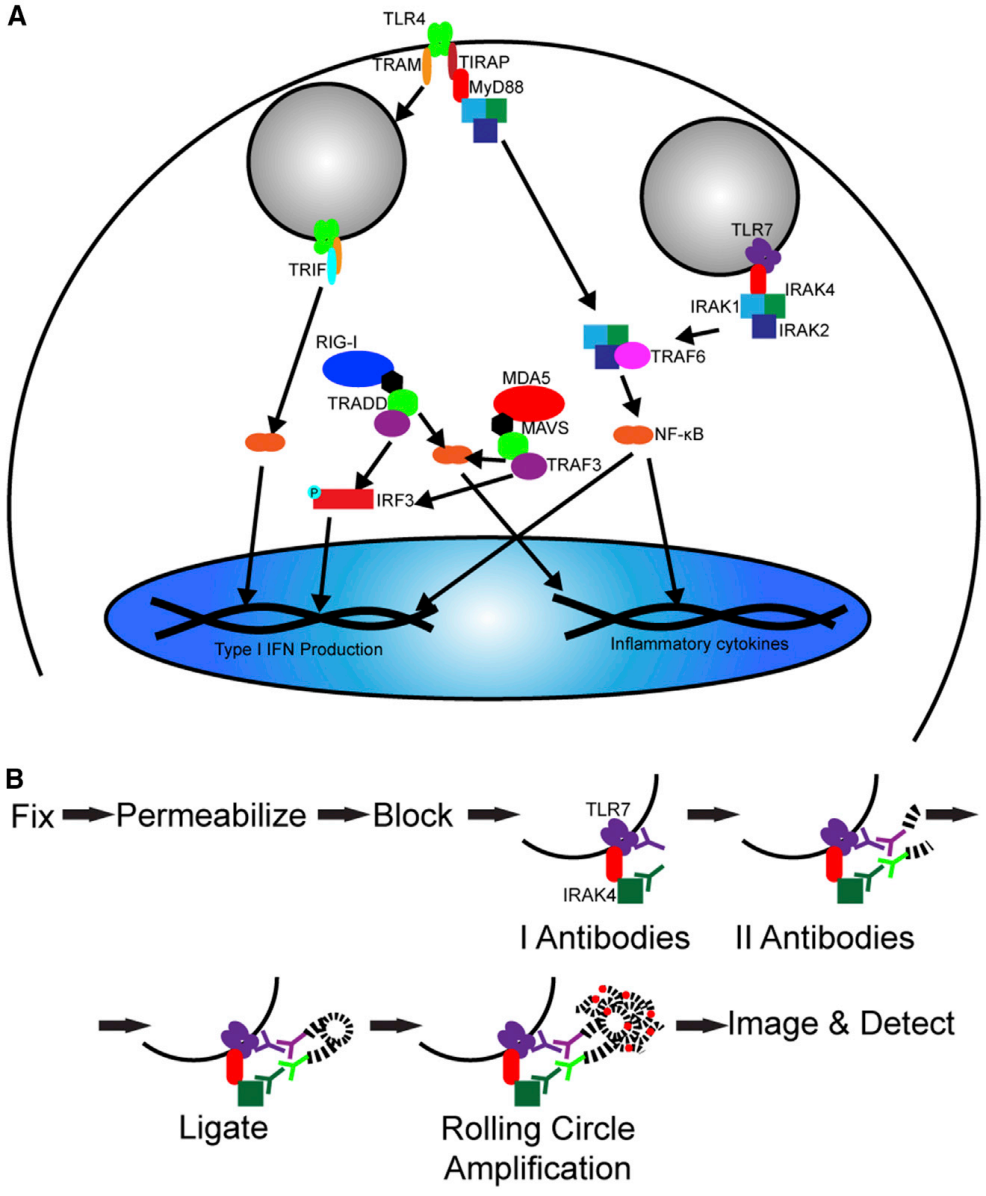
PRR Activation Pathways and PLA Experimental Design (A) Schematic representation of relevant TLR7, RIG-I, MDA5, and TLR4 immune pathways. (B) Schematic representation of PLA methodology.

The primary controllers of the innate immune system are pattern recognition receptors (PRRs). Among these, Toll-like receptors (TLRs) are transmembrane receptors on the cell surface, such as TLR4, which recognizes bacterial wall lipids, or in endosomes, such as TLR7, which detects single-stranded RNA (ssRNA). Cytoplasmic PRRs include retinoic acid-inducible gene I (RIG-I) or melanoma differentiation-associated protein 5 (MDA5), which recognize short double-stranded RNA (dsRNA) with 5′ triphosphorylated (5′ppp) ends or longer dsRNA, respectively. Once activated, PRRs form complexes with adaptor molecules to begin signaling cascades that ultimately lead to type I interferon (IFN) and cytokine induction (Figure 1A).^1^, ^13^, ^14^, ^15^, ^16^, ^17^, ^18^

ELISAs or intracellular immunofluorescence is traditionally used to assess PRR activation by measuring downstream type I IFN and inflammatory cytokines synthesis.^2^, ^6^, ^14^, ^19^, ^20^ However, they cannot identify specific PRR activation, since multiple pathways can lead to similar cytokine induction and crosstalk is common.^18^, ^20^ Additionally, these assays prevent kinetics studies and information on cell-to-cell variability. Lastly, most downstream measurements do not generate spatial information of PRR activation in tissues. For vaccine development, activation in lymph nodes is crucial to immunologic memory.^21^, ^22^ Therefore, determining if PRR activation occurs only in proximity to the delivery site or also in draining lymph nodes is critical. Furthermore, it is valuable to determine if activation is a result of direct interface with the vaccine or from paracrine or endocrine signaling. Alternatively, PRR-knockout (KO) systems can isolate single PRRs. However, they are often complicated to generate or not available, and, most importantly, they cannot assess PRR activation in non-human primate or human studies.^23^

To overcome these limits, we used a proximity ligation assay (PLA) to detect PRR activation in a sensitive, kinetic, and quantitative manner by measuring the formation of specific PRR complexes. While some proteins are ubiquitous across pathways, the complexes they form are unique to each pathway. The formation of such complexes is typically measured by immunoprecipitation, which lacks cell-to-cell variability or spatial information.^24^ In PLA, the proteins of interest are recognized by primary antibodies, which are then recognized by detection antibodies conjugated to specific oligonucleotides (Figure 1B). If the proteins are within 40 nm, the addition of a ligase and complementary oligonucleotides forms a circle, which is amplified by rolling circle amplification. The amplification product is detected *in situ* with fluorescent oligonucleotides. PLA is sensitive, due to the need of close proximity, and quantitative, as the PLA puncta are measurable. PLA can be used in tissue sections, which is critical for clinical studies.^25^, ^26^ We have formerly demonstrated the use of PLA to measure protein-protein interactions, including MDA5 and mitochondrial antiviral-signaling protein (MAVS), as well as protein-RNA interactions.^27^, ^28^, ^29^, ^30^ PLA has also been successfully used to profile cell-signaling pathways and measure cytokine production and receptor dimerization.^31^, ^32^, ^33^ However, none of these previous studies used PLA to look at the early stages of PRR activation, especially in an *in vivo* context.

Here we applied PLA to the study of immune responses elicited by an *in vitro*-transcribed (IVT) mRNA vaccine model, in both a cellular model and *in vivo* mouse studies. IVT mRNA encodes for the antigenic protein(s) and is concurrently detected by anti-viral PRRs, which activate innate immune responses, facilitating the development of protective immune responses.^17^, ^18^, ^19^, ^34^, ^35^, ^36^, ^37^, ^38^, ^39^ By pre-labeling IVT mRNA with multiply labeled tetravalent RNA imaging probes (MTRIPs), its distribution can be assessed by confocal microscopy without affecting metabolism, localization, or translatability.^30^, ^40^ The colocalization of mRNA and PLA signals allows the correlation of immune responses in cells and tissue with downstream immune function. We strongly believe that this approach provides a valuable tool to assess the effect of adjuvants, vaccines, and formulations both *in vitro* and *in vivo* during development and screening.

## Results

### PLA Specifically Detects the Activation of Unique PRR Pathways *In Vitro*

PLA’s efficiency and specificity in detecting PRR activation pathways were first assessed *in vitro* by quantifying TLR7, RIG-I, MDA5, or TLR4 complex formation. Upon activation, TLR7 complexes with myeloid differentiation response gene 88 (MyD88). MyD88 then interacts with the interleukin-1 receptor-associated kinase proteins (IRAK1, IRAK2, and IRAK4), forming the myddosome and beginning a pathway that leads to type I IFN and inflammatory cytokine induction. TLR4 can associate with MyD88 and TIR-domain-containing adaptor protein (TIRAP) and follow similar pathways to lead to inflammatory cytokine production. TLR4 also induces type I IFN through a MyD88-independent pathway by associating with TIR-domain-containing-adaptor protein-inducing IFN-β (TRIF) and TRIF-related adaptor molecule (TRAM).^1^, ^2^, ^4^, ^5^, ^13^, ^14^, ^15^, ^16^, ^17^, ^41^ For cytoplasmic PRRs, both RIG-I and MDA5 associate with interferon-beta promoter stimulator 1 (IPS-1), also known as MAVS, leading to the induction of type I IFN and inflammatory cytokines (Figure 1A).^1^, ^5^, ^13^, ^17^, ^18^ As a result, we chose to quantify the following interactions to distinguish between the PRR pathways: TLR7-IRAK4, RIG-I-MAVS, MDA5-MAVS, TLR4-TIRAP, and TLR4-TRAM.

**Figure 2:**
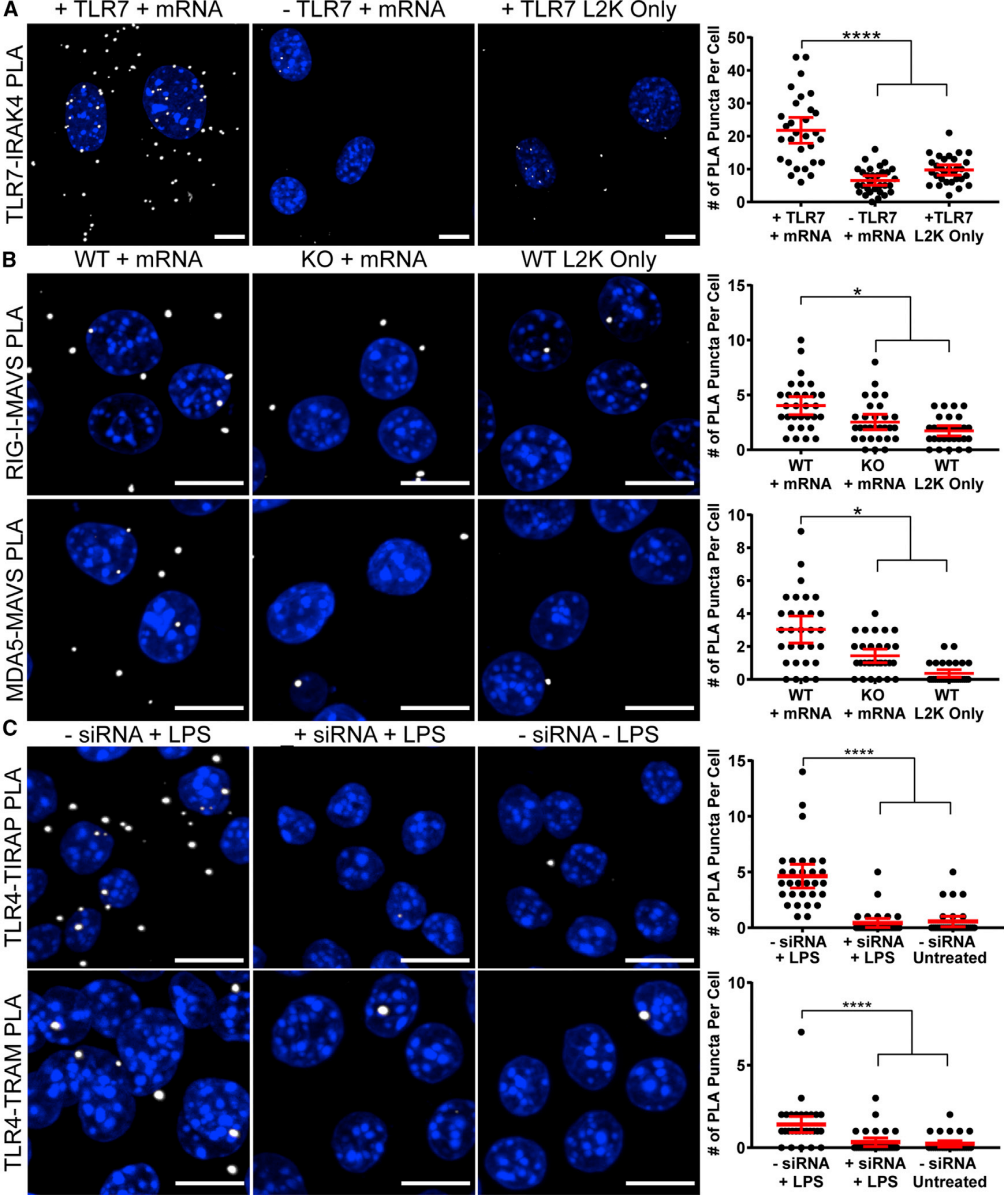
PLA Detection of PRR Activation in Cells with Modulated PRR Expression Levels (A) NIH/3T3 cells were transfected with or without TLR7 via electroporation. After 48 hr, cells were transfected with or without 2.5 μg luciferase mRNA by Lipofectamine 2000 (L2K). Cells were fixed after 24 hr with 1% paraformaldehyde, and PLA was performed between TLR7 and IRAK4. Representative images of PLA (white) and quantification of PLA are shown. Extended focus images are shown. Scale bars, 10 μm. Statistics were performed with a one-way ANOVA with a Dunn’s multiple comparisons test, where n = 30 and ****p < 0.0001. 95% confidence intervals are shown in red. (B) RAW-Lucia ISG macrophages, WT, RIG-I KO, or MDA5 KO, were transfected with or without 2.5 μg luciferase mRNA by Lipofectamine 2000. Cells were fixed after 24 hr with ice-cold methanol, and PLA was performed between RIG-I and MAVS or MDA5 and MAVS. Representative images of PLA (white) and quantification of PLA are shown. Extended focus images are shown. Scale bars, 10 μm. Statistics were performed with a one-way ANOVA with a Dunn’s multiple comparisons test, where n = 30 and *p < 0.035. 95% confidence intervals are shown in red. (C) RAW 264.7 macrophages were transfected with or without 100 nM TLR4 siRNA via Lipofectamine 2000. After 24 hr, cells were incubated with either 1 or 10 μg/mL LPS for TIRAP and TRAM interactions, respectively. After 30 min, cells were fixed with 1% paraformaldehyde, and PLA was performed between TLR4 and TIRAP and TLR4 and TRAM. Representative images of PLA (white) and quantification of PLA are shown. Extended focus images are shown. Scale bars, 10 μm. Statistics were performed with a one-way ANOVA with a Dunn’s multiple comparisons test, where n = 30 and ****p < 0.0001. 95% confidence intervals are shown in red.

Activation of TLR7, RIG-I, and MDA5 was induced via IVT mRNA transfection. As TLR4 is not stimulated by nucleic acids, lipopolysaccharide (LPS) was used as an agonist. PRR activation was quantified in wild-type (WT) cells and cells expressing modulated PRR levels. TLR7 PLA was evaluated in NIH/3T3 cells, which naturally express low levels of TLR7, and in cells transfected with a TLR7-encoding plasmid. RIG-I and MDA5 activation was assayed in RAW-Lucia ISG macrophages, using either the WT cell line or the commercially available RIG-I- or MDA5-KO cell lines. As TLR4-KO cells were not readily available, small interfering RNA (siRNA) was used to knock down TLR4 in RAW 264.7 macrophages for comparison with WT cells. Lipofectamine-only controls were included in each condition. TLR7-IRAK4 interactions were significantly higher in cells that were both stimulated by IVT mRNA and that expressed high levels of TLR7, in comparison to cells with basal levels of TLR7 or unstimulated cells (Figure 2A). Similar results were observed for RIG-I and MDA5 interactions with MAVS (Figure 2B). In both assays, WT cells stimulated with IVT mRNA generated a significantly higher number of interactions than KO cells or unstimulated WT cells. Accordingly, cells treated with TLR4 siRNA displayed significantly fewer interactions with TIRAP or TRAM (Figure 2C). These results demonstrated the specificity of the PLA methodology to detect PRR activation through the quantification of PRR protein complex formation.

### PLA Sensitively Detects Kinetic Changes in PRR Pathway Activation *In Vitro*

**Figure 3:**
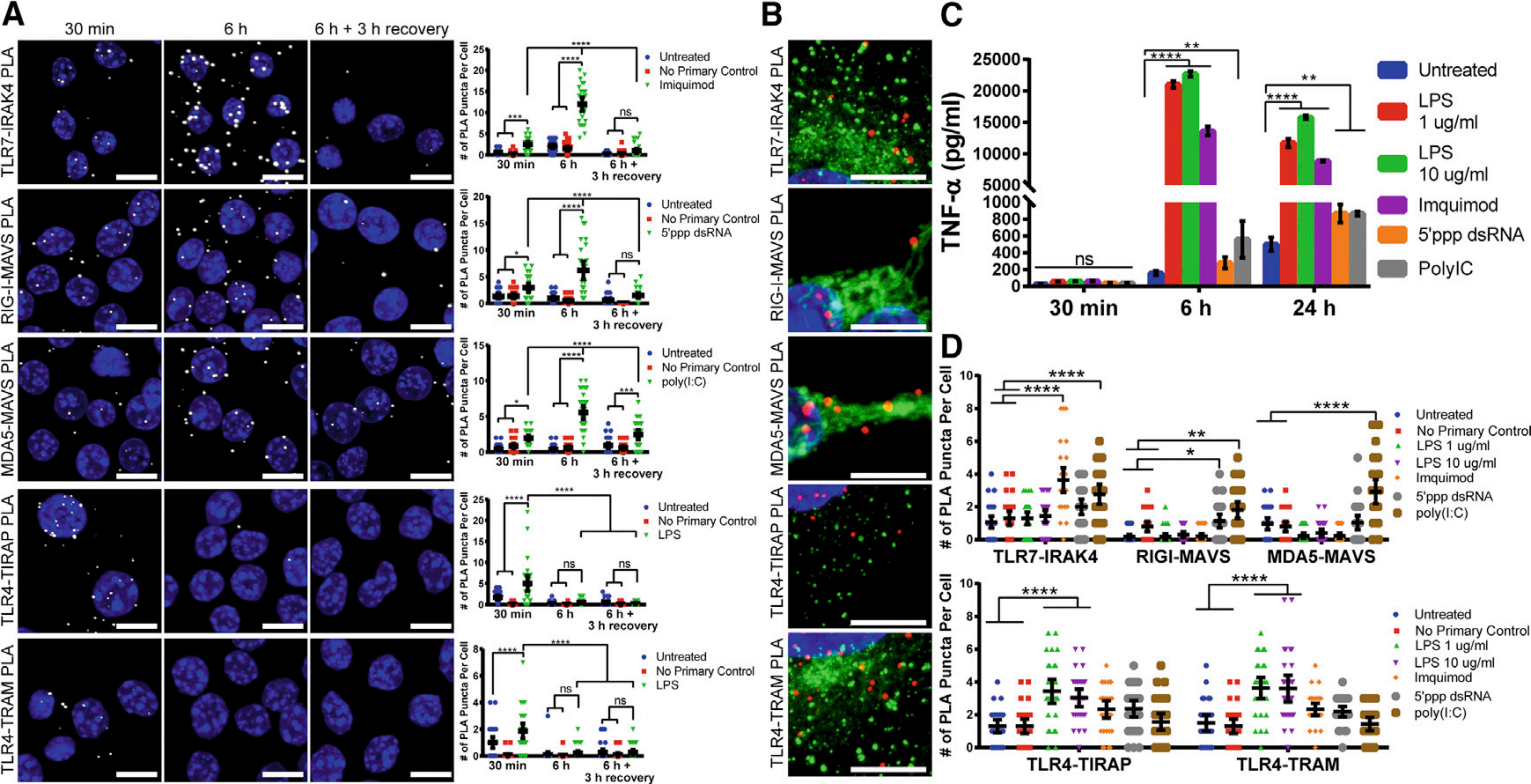
PLA Sensitively Detects PRR Activation Kinetics (A) RAW 264.7 macrophages were incubated with the following agonists: 8 μg/mL imiquimod for TLR7, 10 μg/mL 5′ppp dsRNA for RIG-I, 0.1 μg/mL poly(I:C) for MDA5, 1 μg/mL LPS for TLR4-TIRAP, and 10 μg/mL LPS for TLR4-TRAM. Cells were fixed with 1% paraformaldehyde or methanol as previously indicated after 30 min or 6 hr. Also at 6 hr, agonists were removed and cells were incubated with media for 3 additional hours before fixing. PLA was performed between TLR7-IRAK4, RIG-I-MAVS, MDA5-MAVS, TLR4-TIRAP, and TLR4-TRAM. Representative images of PLA (white) and quantification of PLA are shown. Extended focus images are shown. Scale bars, 10 μm. Statistics were performed with a two-way ANOVA with a Tukey’s multiple comparisons test, where n = 30 and *p < 0.031, ****p < 0.0007, and ****p < 0.0001. 95% confidence intervals are shown in black. (B) A549 cells for TLR7-IRAK4, TLR4-TIRAP, and TLR4-TRAM PLAs were transfected with CellLight Early Endosomes-GFP-BacMam 2.0 for 16 hr prior to stimulation with 8 μg/mL imiquimod for TLR7 or 1 μg/mL LPS for TLR4-TIRAP and 10 μg/mL LPS for TLR4-TRAM for 30 min before fixation with 1% paraformaldehyde. RAW 264.7 macrophages for RIG-I-MAVS and MDA5-MAVS PLAs were incubated with MitoTracker for 15 min prior to stimulation with 0.1 μg/mL poly(I:C) for RIG-I or MDA5 for 30 min before fixation with methanol. PLA was performed between TLR7-IRAK4, RIG-I-MAVS, MDA5-MAVS, TLR4-TIRAP, and TLR4-TRAM. Representative images of PLA (red) and either endosomes (green) (TLR7-IRAK4, TLR4-TIRAP, and TLR4-TRAM) or mitochondria (green) (RIG-I-MAVS and MDA5-MAVS) are shown. Extended focus images are shown. Scale bars, 10 μm. (C and D) RAW 264.7 macrophages were incubated with the following agonists: 8 μg/mL imquimod, 10 μg/mL 5′ppp dsRNA, 0.1 μg/mL poly(I:C), and 1 or 10 μg/mL LPS. Supernatants were collected, and cells were fixed with 1% paraformaldehyde or methanol as previously indicated after 30 min or 6 or 24 hr. (C) An ELISA for TNF-α was performed on supernatants. Quantification is shown with SDs in black. Statistics were performed with a two-way ANOVA with a Tukey’s multiple comparisons test, where n = 2 and *p < 0.007 and ****p < 0.0001. (D) PLA was performed at the 30-min time point for TLR7-IRAK4, RIG-I-MAVS, MDA5-MAVS, TLR4-TIRAP, and TLR4-TRAM. Quantification is shown, with 95% confidence intervals in black. Statistics were performed with a two-way ANOVA and a Tukey’s multiple comparisons test, where n = 30 and *p < 0.01, **p < 0.007, and ****p < 0.0001.

We subsequently evaluated PLA’s ability to sensitively measure PRR activation kinetics. Well-characterized soluble PRR agonists were delivered to RAW 264.7 macrophages, and PRR activation-induced complexes were measured over several time points. 8 μg/mL imiquimod was used to activate TLR7, 10 μg/mL 5′ppp dsRNA was used to activate RIG-I, while 0.1 μg/mL polyinosinic:polycytidylic acid [poly(I:C)] was utilized as an MDA5 agonist. 1 μg/mL LPS was used to stimulate the TLR4-TIRAP pathway, while 10 μg/mL LPS was used to stimulate the TLR4-TRAM pathway. To assess kinetics, PRR activation was measured at 30 min and 6 hr after delivery of the agonists. Additionally, at 6 hr, the agonists were removed, and the cells were incubated with fresh media for 3 hr to allow for the recovery of base levels of PRR activation. PRR activation was detected for TLR7, RIG-I, MDA5, and both TLR4 pathways as early as 30 min after agonist delivery (Figures 3A and S1). At 6 hr post-delivery, the number of interactions significantly increased for TLR7, RIG-I, and MDA5, indicating that an amplified PRR response occurred as time progressed. Finally, when the agonists were removed and the cells were allowed to recover, the number of interactions for TLR7, RIG-I, and MDA5 significantly decreased, with TLR7 and RIG-I displaying no significant activation. TLR4 interactions with TIRAP or TRAM decreased 6 hr post-delivery and were not affected by recovery. These results demonstrated PLA’s sensitivity in detecting the expected changes of activation over time in response to known agonists.

By fluorescently labeling organelles prior to PLA, we demonstrated the colocalization of PLA puncta with endosomes and mitochondria (Figure 3B). TLR7-IRAK4 and TLR4-TRAM interactions occur on endosomal membranes; accordingly, PLA puncta were visualized immediately adjacent to endosomes. Conversely, PLA puncta for TLR4-TIRAP interactions, which occur on the cell membrane, did not colocalize with endosomes. MAVS is localized on the mitochondria membranes; accordingly, RIG-I-MAVS and MDA5-MAVS interactions colocalized with mitochondria staining.^42^ It is important to note that PLA puncta are generated by rolling circle amplification, which results in the synthesis of a long DNA chain. Consequently, the puncta can slightly diffuse around the origin point (the location of the interacting protein pair), so perfect colocalization with endosomes or mitochondria was not expected. These results validated that the PLA method successfully detects the indicated interactions in proximity to the expected cellular compartments.

Next, we compared PRR activation by PLA to tumor necrosis factor alpha (TNF-α) production by ELISA. RAW 264.7 macrophages were stimulated with 8 μg/mL imiquimod, 10 μg/mL 5′ppp dsRNA, 0.1 μg/mL poly(I:C), and 1 or 10 μg/mL LPS. Supernatants were collected and cells were fixed at 30 min or 6 or 24 hr post-stimulation. An ELISA was performed for each time point (Figure 3C). TNF-α production by ELISA was detected at 6 and 24 hr, while no TNF-α was detected at 30 min. Importantly, all agonists stimulated TNF-α release. PRR activation was detected by PLA as early as 30 min post-treatment (Figures 3A and 3D).

Critically, only the expected agonists triggered specific pathways when measured by PLA, with the exception of TLR7-IRAK4, whose activation was also observed upon the delivery of poly(I:C), likely due to the transfection agent needed for its cellular entry. These data demonstrate that PLA is superior to conventional methods, such as ELISAs, due to its ability to isolate specific pathways, as well as to detect early activation kinetics.

### PLA Detects Differences in PRR Pathway Activation Induced by Modified Nucleosides of IVT mRNA

**Figure 4:**
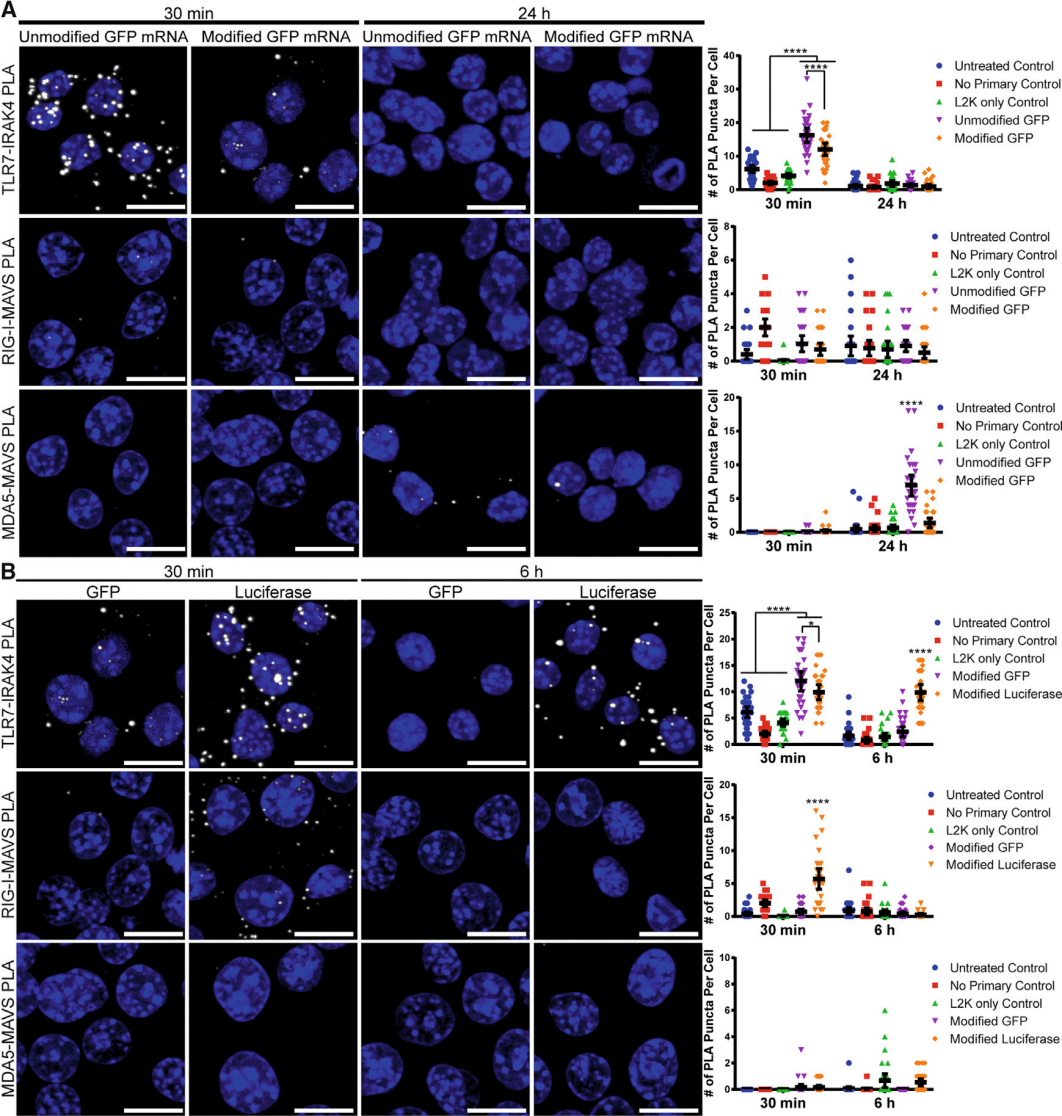
Differences in PRR Activation between Unmodified and Modified IVT mRNAs and between mRNA Sequences (A) RAW 264.7 macrophages were untreated, transfected with Lipofectamine 2000 (L2K) alone, or transfected with 200 ng unmodified or modified IVT GFP mRNA via L2K. Cells were fixed with either 1% paraformaldehyde or methanol after 30 min or 24 hr. Representative images of PLA (white) and quantification of PLA puncta per cell are shown. Extended focus images are shown. Scale bars, 10 μm. Statistics were performed with a two-way ANOVA with a Tukey’s multiple comparisons test, where n = 30 and ****p < 0.0001. 95% confidence intervals are shown in black. (B) RAW 264.7 macrophages were untreated, transfected with Lipofectamine 2000 (L2K) alone, or transfected with 200 ng modified IVT GFP or luciferase mRNA via L2K. Cells were fixed with either 1% paraformaldehyde or methanol after 30 min or 6 hr. Representative images of PLA (white) and quantification of PLA are shown. Extended focus images are show. Scale bars, 10 μm. Statistics were performed with a two-way ANOVA with a Tukey’s multiple comparisons test, where n = 30 and *p < 0.045 and ****p < 0.0001. 95% confidence intervals are shown in black.

We next explored PLA’s sensitivity in quantifying innate immune responses to delivered IVT mRNA. It has been shown that incorporating modified nucleosides into IVT mRNA reduces PRR activation.^10^, ^11^ Therefore, we quantified PRR activation via PLA upon delivery of either unmodified or modified IVT GFP mRNA. mRNA was transfected into RAW 264.7 macrophages with Lipofectamine 2000, and cells were fixed at either 30 min or 24 hr post-delivery. While neither mRNA activated a RIG-I response, unmodified mRNA induced a significantly higher TLR7 activation at 30 min and MDA5 activation at 24 hr (Figures 4A and S2). This activation pattern is expected, due to the entry pathway of mRNA via Lipofectamine, which proceeds through the endocytic compartment. Therefore, the mRNA interacts with membrane-bound PRRs first, is internalized in endocytic vesicles, and is then released into the cytosol, where it interacts with cytoplasmic PRRs such as RIG-I and MDA5.^43^, ^44^, ^45^ This demonstrates PLA’s ability to detect the changes in PRR activation due to base modifications in IVT mRNA. It should be noted that TLR4 activation was not measured, as mRNA is not one of its agonists.

### PLA Measures the Difference in PRR Pathway Activation between IVT mRNA Sequences

We also examined PLA’s sensitivity in quantifying PRR activation due to different IVT mRNA sequences. GFP- or luciferase-encoding IVT mRNA, both containing modified nucleosides but with different 3′ UTRs and coding regions, was delivered to RAW 264.7 macrophages with Lipofectamine 2000 and fixed after 30 min and 6 hr. Differences in IFN-α production between GFP and luciferase mRNAs have previously been discussed in the literature with both unmodified and modified mRNAs.^46^ GFP mRNA induced a fast and strong TLR7 response at 30 min, which ceased by 6 hr post-delivery. Conversely, luciferase mRNA induced a strong TLR7 response that persisted up to 6 hr post-delivery and a rapid, strong RIG-I response at 30 min post-transfection (Figures 4B and S2). Overall, these data indicate that different mRNAs induce the activation of alternative PRR pathways, which can be specifically identified and monitored using PLA.

### PLA Detects PRR Pathway Activation *In Vivo* in Muscle Tissue

Next, we aimed to quantify PRR activation via PLA in muscle tissue after intramuscular delivery of mRNA in mice. To visualize mRNA distribution in the muscle, IVT mRNA encoding for firefly luciferase was pre-labeled with MTRIPs targeting three regions within the 3′ UTR (Figures S3A and S3B). MTRIP labeling did not affect PRR activation upon transfection in RAW 264.7 cells with modified or unmodified IVT GFP mRNA (Figure S4).

IVT mRNA was injected intramuscularly (i.m.) either naked or formulated with the nanoparticle CholK, for improved delivery, especially to lymph nodes over naked delivery.^40^ At 1.5 hr following injection, IVT mRNA delivered alone or complexed to CholK was evenly distributed throughout the muscle, primarily between skeletal muscle fibers. At 16 hr following injection, naked IVT mRNA levels decreased significantly in the muscle, potentially as a consequence of mRNA degradation, while mRNA complexed with CholK remained present throughout the tissue (Figure S5A). We assessed expression of TLR7, RIG-I, and MDA5 via immunofluorescence staining in skeletal muscle tissue following the injection with either naked IVT mRNA or a sham control. Upregulated PRR expression was observed via immunofluorescence in these tissues 16 hr following the injection of fluorescently labeled naked IVT mRNA, in comparison to sham-injected muscle (Figures S6 and S7). We observed that cells with elevated PRR levels were distributed throughout the skeletal muscle tissue and that these cell types were associated with IVT mRNA. To assess the uptake of IVT mRNA into immune cells 16 hr following injection, flow cytometry was performed. IVT mRNA injection led to an increased presence of CD11b^+^ cells in the muscle (Figure S5B). Mice injected with IVT mRNA complexed with CholK also showed increased levels of MHCII^+^ cells, as well as infiltration of natural killer cells, identified by the dual staining of CD335 and CD11b.

Based on the upregulated expression of PRRs as evidenced by immunofluorescence, as well as by the uptake of mRNA by infiltrating immune cells, we next examined the PRR response via PLA at 1.5, 6, and 16 hr following i.m. injection of fluorescently labeled IVT mRNA, either naked or complexed with CholK, as well as after a sham injection. We noticed that, in tissue sections, PRR activation did not result in the formation of single PLA puncta but rather in large aggregates. Therefore, PRR activation was quantified by measuring the total PLA signal volume.

**Figure 5:**
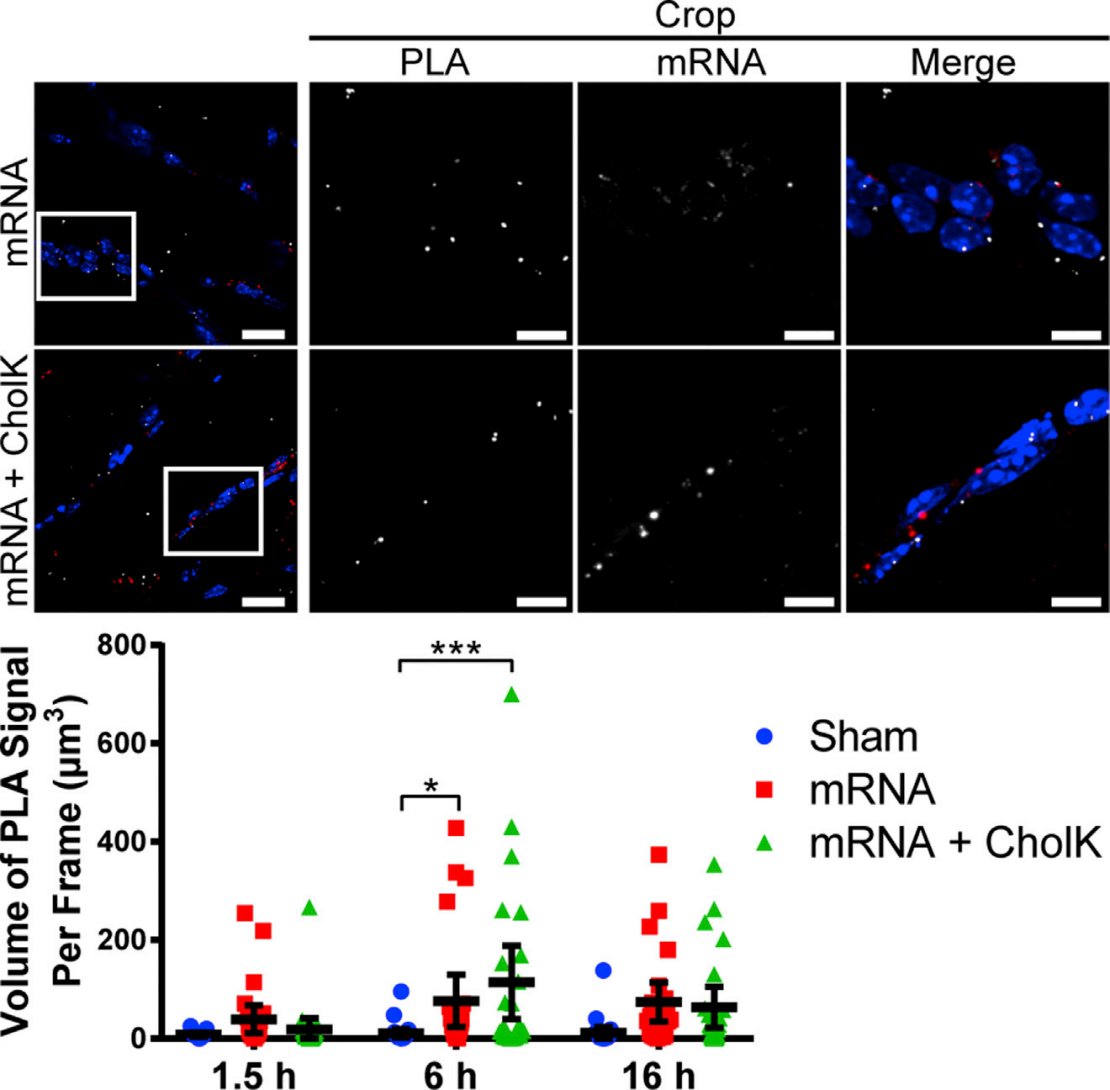
TLR7 Activation by PLA in Muscle Tissue Representative images of TLR7-IRAK4 PLA (white) 6 hr following i.m. injection into the anterior tibialis of 10 μg luciferase IVT mRNA (red) unformulated or formulated with CholK. Extended focus images are shown. Scale bars, 20 μm (full images) and 5 μm (crops). White boxes show cropped selection. Quantification of TLR7-IRAK4 PLA is shown. Statistics were performed with a two-way ANOVA with a Tukey’s multiple comparisons test, where n = 30, except for sham 1.5 hr where n = 12, and *p < 0.04 and ***p < 0.0004. 95% confidence intervals are shown in black.

**Figure 6:**
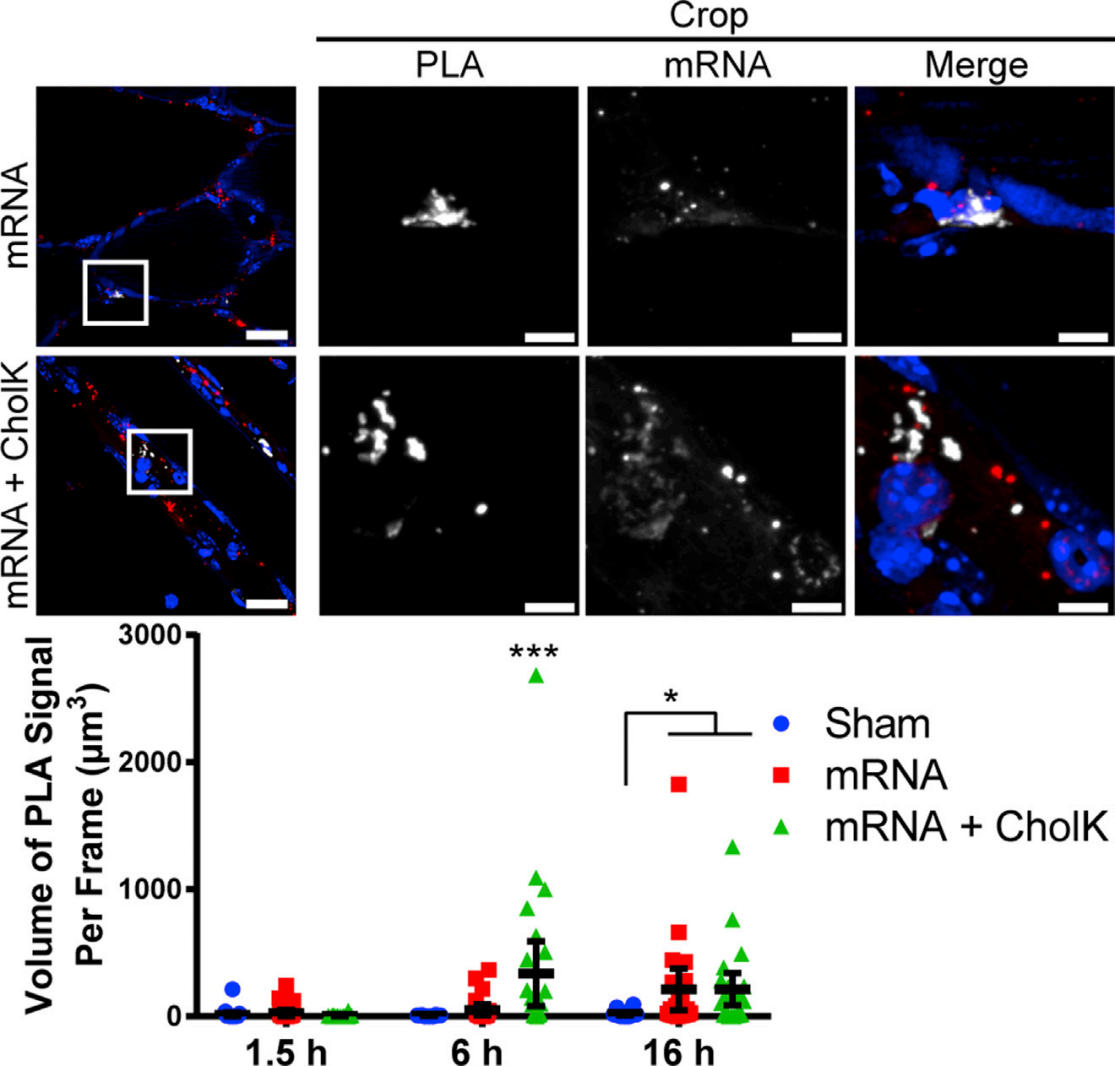
RIG-I Activation by PLA in Muscle Tissue Representative images of RIG-I-MAVS PLA (white) 6 hr following i.m. injection into the anterior tibialis of 10 μg luciferase IVT mRNA (red) unformulated or formulated with CholK. Extended focus images are shown. Scale bars, 20 μm (full images) and 5 μm (crops). White boxes show cropped selection. Quantification of RIG-I-MAVS PLA is shown. Statistics were performed with a two-way ANOVA with a Tukey’s multiple comparisons test, where n = 30 and *p < 0.03 and ***p < 0.0007. 95% confidence intervals are shown in black.

**Figure 7:**
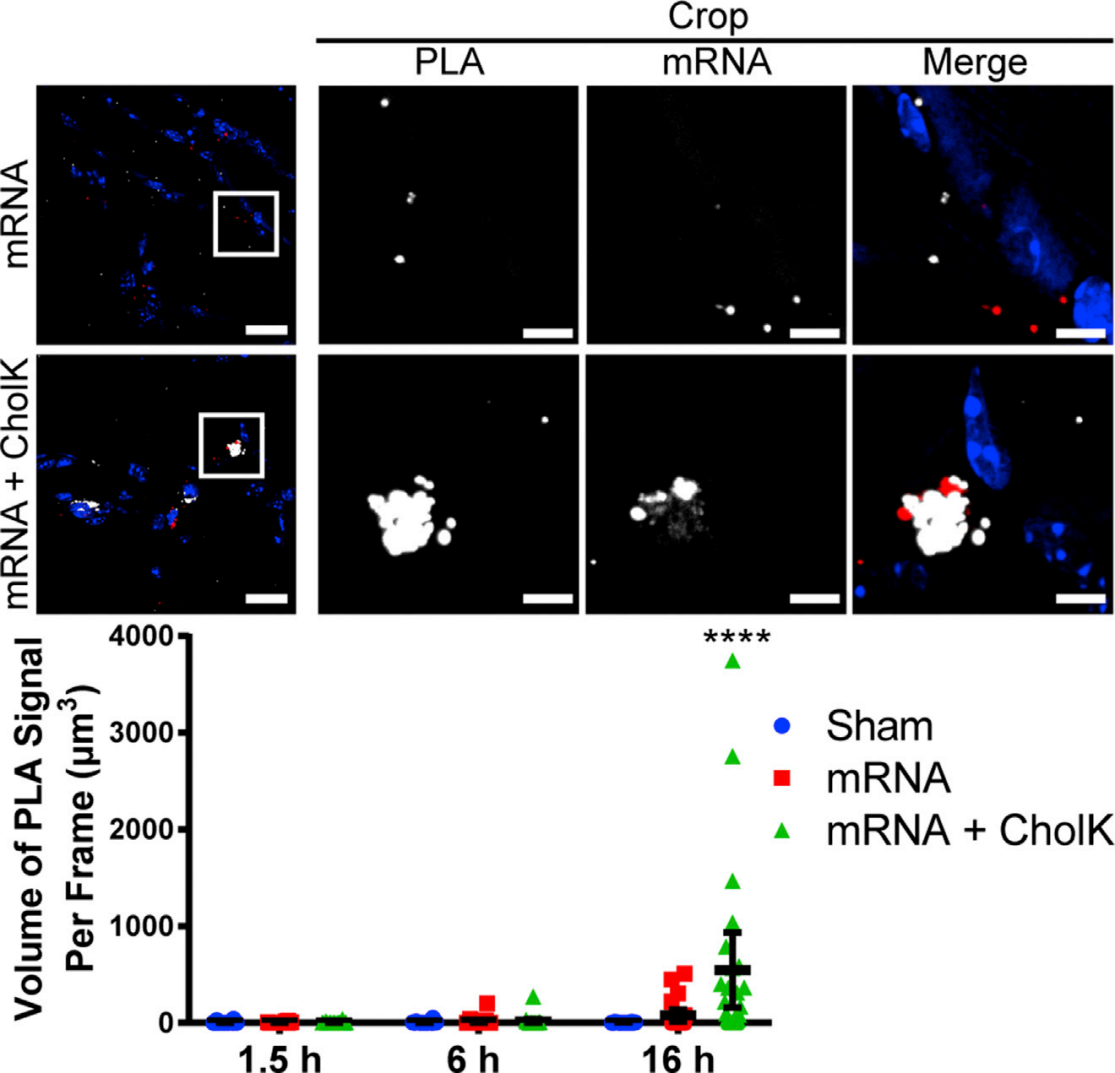
MDA5 Activation by PLA in Muscle Tissue Representative images of MDA5-MAVS PLA (white) 16 hr following i.m. injection into the anterior tibialis of 10 μg luciferase IVT mRNA (red) unformulated or formulated with CholK. Extended focus images are shown. Scale bars, 20 μm (full images) and 5 μm (crops). White boxes show cropped selection. Quantification of MDA5-MAVS PLA is shown. Statistics were performed with a two-way ANOVA with a Tukey’s multiple comparisons test, where n = 30 and ****p < 0.0001. 95% confidence intervals are shown in black.

Naked mRNA induced significant TLR7 activation at 6 hr and RIG-I activation at 16 hr post-delivery, respectively (Figures 5 and 6; Figure S8). mRNA formulated with CholK induced a significant TLR7 activation at 6 hr, RIGI activation at 6 and 16 hr, and MDA5 activation at 16 hr post-delivery (Figures 5, 6, and 7; Figure S8), consistent with the observed prolonged retention of the IVT mRNA within tissue (Figure S5A). PLA signal was clearly observed in close proximity to delivered mRNA, demonstrating that the PRR activation occurred primarily in cells that internalized mRNA and not uniformly across the tissue.

### PLA Detects PRR Activation *In Vivo* in Lymph Nodes after i.m. Injection

**Figure 8:**
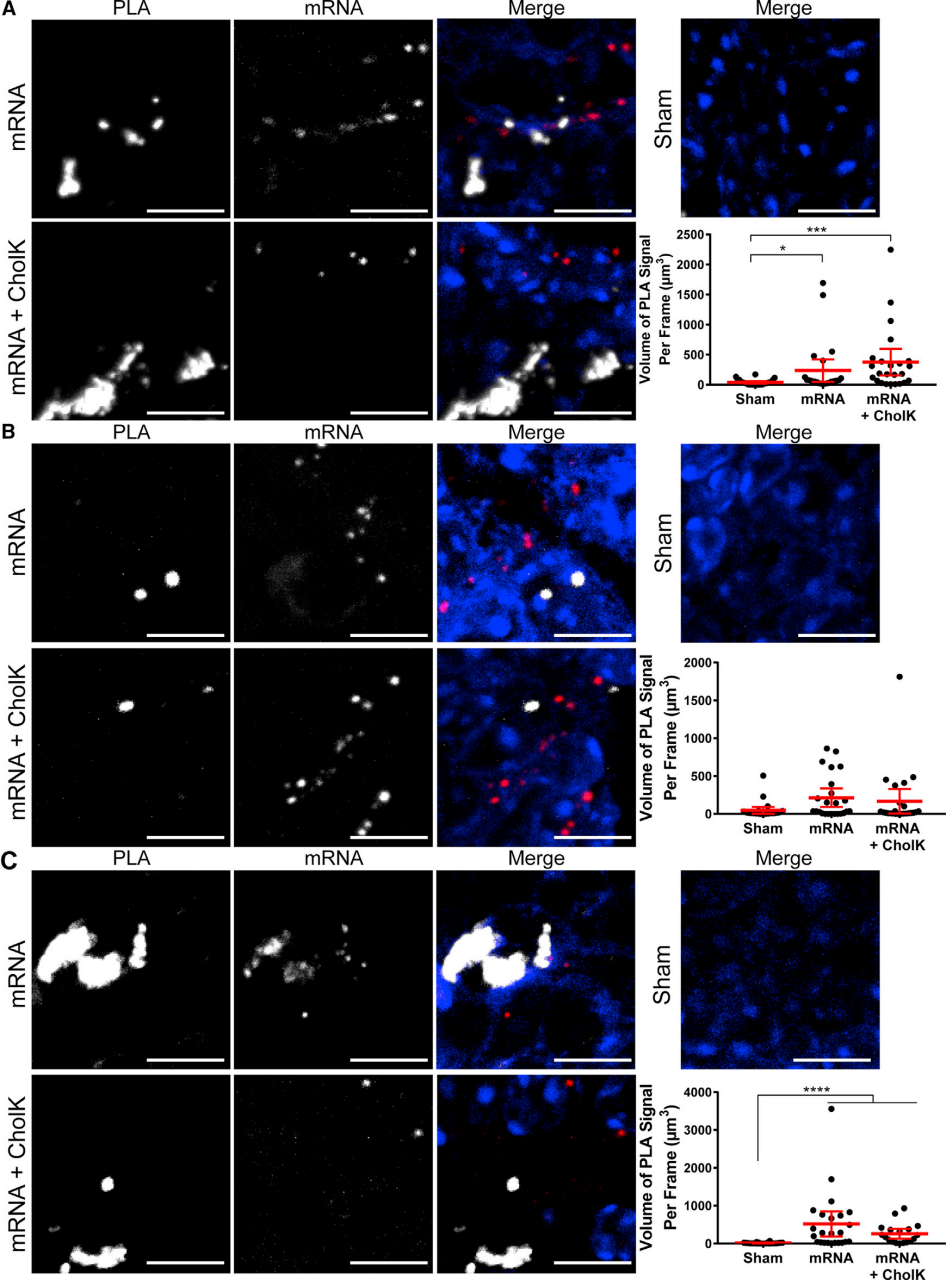
PRR Activation by PLA in Lymph Node Tissue Lymph nodes were excised and fixed 16 hr following i.m. injection into the anterior tibialis of 10 μg luciferase IVT mRNA (red) unformulated or formulated with CholK. (A) Representative images of TLR7-IRAK4 PLA (white) shown. Extended focus images are shown with 10-μm scale bars. Quantification of TLR7-IRAK4 PLA is shown. Statistics were performed with a one-way ANOVA with a Dunn’s multiple comparisons test, where n = 30 and *p < 0.02 and ***p < 0.0002. 95% confidence intervals are shown in red. (B) Representative images of RIG-I-MAVS PLA (white) shown. Extended focus images are shown with 10 μm scale bars. Quantification of RIG-I-MAVS PLA is shown. Statistics were performed with a one-way ANOVA with a Dunn’s multiple comparisons test, where n = 30. 95% confidence intervals are shown in red. (C) Representative images of MDA5-MAVS PLA (white) shown. Extended focus images are shown with 10 μm scale bars. Quantification of MDA5-MAVS PLA is shown. Statistics were performed with a one-way ANOVA with a Dunn’s multiple comparisons test, where n = 30, except for the mRNA + CholK condition where n = 18, and ****p < 0.0001. 95% confidence intervals are shown in red.

Following i.m. injection of fluorescently labeled IVT mRNA, the Fluobeam imaging system (Fluoptics) was used on euthanized mice to determine IVT mRNA trafficking to the draining lymph nodes (Figure S9) and, specifically, to the lumbar aortic, inguinal, and popliteal lymph nodes. Lymph nodes were histologically analyzed to assess IVT mRNA distribution (Figures S10A–S10C). In tissue sections, IVT mRNA was observed around and within the lymphatic vessels stained for Lyve-1. Isolated lymph nodes were homogenized to identify the cell types positive for IVT mRNA based on their cell surface markers by flow cytometry (Figure S10D). IVT mRNA was largely present in antigen-presenting cells, such as MHCII^+^, CD11b^+^, and CD19^+^CD3^−^ (B cell) lymph node cells. Next, PRR activation was assessed in the lumbar aortic lymph nodes excised 16 hr post-injection (Figure 8). Both naked and CholK-delivered IVT mRNA significantly induced the activation of TLR7 and MDA5. These results highlight PLA versatility in detecting PRR activation across multiple tissues types.

## Discussion

Here we demonstrated the use of PLA as a sensitive, quantitative, and specific tool to measure PRR activation in cells and in tissue upon IVT mRNA delivery. First, we compared PRR activation with modulated protein levels *in vitro*, as well as over time in response to known agonists. This validated the specificity of PLA to detect individual PRR pathways, as interactions unique to each pathway were identified and quantified, indicating both the antibodies’ specificity for the target complex molecules and the assay’s specificity for complex formation. Furthermore, this demonstrated the versatility of PLA, as it was successfully optimized for several PRR pathways whose activation occurred in distinct cellular compartments: TLR4 on the cell surface membrane, TLR7 in the endosome, and RIG-I and MDA5 in the cytoplasm. Immunofluorescence colocalization of PLA puncta with cellular organelles further verified the ability of PLA to distinguish between pathways in different cellular compartments. This provides support to the translatability of PLA to detect other PRR pathways not detailed in this work.

PLA was also shown to be able to distinguish TLR4 sub-pathways, confirming its ability to isolate unique activation routes. Moreover, PLA successfully detected activation kinetics and quantitatively measured the turning on and turning off of individual PRR pathways *in vitro*. This sensitivity is critical because the formation of these complexes is transient and unique to each pathway. PLA was able to specifically identify the individual pathway, as interactions for each pathway were not detected if non-expected agonists were used. Furthermore, the results support the sensitivity of PLA to distinguish levels of PRR activation over time at earlier time points than ELISAs. Comparing the strength of responses induced by two candidate agonists, for example, would provide critical information during vaccine and therapeutic development.

Subsequently, we examined PLA’s suitability in measuring the PRR response of synthetic IVT mRNAs. We compared unmodified mRNA to mRNA with modified nucleosides previously seen to be immunomodulatory.^10^, ^11^ These results confirm PLA can be used to screen IVT mRNA for PRR activation, as it confirms the previously reported modulatory effect of modified nucleosides in IVT mRNA. The difference in activation times between the PRRs is most likely due to the delivery method. As Lipofectamine prompts internalization via endosomes,^43^, ^44^, ^45^ the IVT mRNA interacts with TLR7 earlier than MDA5, which is cytoplasmic and first requires mRNA escape from the endosomal compartment.

We also demonstrated that PLA can measure differences in PRR activation induced by two model reporter mRNAs. We confirmed that mRNA sequence is a determinant in PRR activation, supporting differences in IFN-α production previously observed in the literature.^46^ Despite delivering equivalent amounts of mRNA, luciferase mRNA was released from the endosome as early as 30 min and induced a significant RIG-I response, unlike GFP mRNA. Moreover, luciferase mRNA was retained in the endosomes longer than GFP mRNA, as it still produced a significant TLR7 response at 6 hr post-delivery. These differences are likely due to the internalization time and secondary structure. Size difference between the mRNAs may cause a difference in charge, affecting the complexation efficiency with Lipofectamine 2000.

Both charge ratio and size of lipoplexes have been observed to affect transfection time and efficiency.^43^, ^47^ Downstream cytokine measurement would be insufficient to compare the persistence of the TLR7 response, as both would produce type I IFN and inflammatory cytokines. PLA, however, was able to detect shifting levels of activation over time. The difference in RIG-I activation is likely due to the formation of unique secondary structures in the mRNAs as a result of their sequences, indicating that the luciferase mRNA may have more prominent secondary structures than GFP mRNA, or due to differences in capping efficiency and phosphatase activity for this particular mRNA.^17^, ^18^, ^48^, ^49^ As a whole, these results demonstrate PLA’s value in screening nucleotide-based therapeutics and vaccines in their developing stage. RNA vaccines for two different diseases may require different delivery formulations to induce the same immune response and protective immunity.

Another advantage of PLA measurements *in vitro* is that they could be performed in a 96-well glass-bottom plate using the manufacturer’s protocol. Several interacting protein pairs can be assessed in the same plate and imaged directly in the plate. The Volocity acquisition software could be set to automatically acquire images in each well, allowing for an automated imaging process. As a result, PLA allows the measurement of PRR activation in a high-throughput screening fashion, establishing it as an ideal approach to quickly characterize agonist-induced PRR activation in cells prior to their use in *in vivo* experiments.

We next demonstrated the use of PLA as a PRR quantification tool *in vivo* in tissue. First, we verified the successful delivery of the IVT mRNA into muscle via i.m. injection, and we assessed the mRNA-induced recruitment of immune cells. CholK-mediated delivery enhanced immune cell infiltration over naked delivery, as supported by flow cytometry and immunofluorescence. Furthermore, mRNA complexed with CholK was retained in the muscle longer than free mRNA, inducing a stronger RIG-I response at 6 hr and a stronger MDA5 activation at 16 hr. We hypothesize that CholK enhanced cytoplasmic delivery of the mRNAs in tissue, causing the stronger cytoplasmic PRR responses of complexed mRNA over naked mRNA, as supported by our prior work.^40^ The extended mRNA retention in the muscle tissue is likely the main cause of MDA5 activation at 16 hr post-injection. Elevated RIG-I and MDA5 activation from complexed mRNA could generate a strong type I IFN response, inducing further immune cell recruitment.^50^ We also observed that PLA signal was localized in close proximity to the mRNA, indicating the PRR activation was a direct result of internalized mRNA rather than a paracrine response.

Type I IFN is also known to promote migration of antigen-presenting cells to lymph nodes.^50^, ^51^ mRNA was detected in the lymph nodes at 16 hr after i.m. injection and resulted in significant TLR7 and MDA5. Though naked mRNA trafficked to the lymph nodes and activated PRRs, the increased infiltration of immune cells in the muscle observed with CholK-formulated mRNA most likely enhanced cell-mediated trafficking of the IVT mRNA, enhancing TLR7 activation. Trafficking of antigens to the lymph node and the subsequent PRR activation is fundamental since the lymph node is the site of adaptive immunity initiation. Observation of PRR activation in lymph nodes is, therefore, critical in vaccine development. Overall, these findings clearly indicate PLA is a versatile tool that can be used across multiple tissues.

In conclusion, we demonstrated PLA’s use as a method of detection of innate immune responses both *in vitro* in cells and in tissue sections. This valuable tool allows for sensitive, specific, and quantitative measurements of the kinetics of PRR activation. PLA can be used in a high-throughput screening manner, to compare the effect of multiple agonists, adjuvants, or therapies easily and rapidly *in vitro*. Finally, it allows insights into the spatial context where the activation occurs, both in single cells and across whole tissues. We believe PLA has the potential to be a crucial tool in therapeutic and vaccine development.

## Materials and Methods

### Antibodies for PLA

Primary antibodies used were as follows: mouse anti-RIG-I (sc-376845, 1:500; Santa Cruz Biotechnology, Dallas, TX), goat anti-MDA5 (sc-48031, 1:500; Santa Cruz Biotechnology, Dallas, TX), rabbit anti-MAVS (IHC-00477, 1:500; Bethyl Laboratories, Montgomery, TX), rabbit anti-TLR7 (NBP2-24906, 1:500; Novus Biologicals, Littleton, CO), mouse anti-IRAK4 (LS BIO452, 1:500; LS Bio, Seattle, WA), rabbit anti-TLR4 (NB100-56581, 1:50; Novus Biologicals, Littleton, CO), goat anti-TIRAP (NB300-990, 1:100; Novus Biologicals, Littleton, CO), and goat anti-TRAM (AF4348, 1:1,000; Novus Biologicals, Littleton, CO).

### *In Vitro* PLA on Cells with Differential TLR7 Expression

NIH/3T3 mouse fibroblast cells (ATCC CRL-1658, Manassas, VA) were maintained in High Glucose DMEM (Lonza, Allendale, NJ) with 20% bovine calf serum (Gibco, Waltham, MA). NIH/3T3 cells do not express detectable levels of TLR7^52^ and were used with or without induced expression of TLR7. NIH/3T3 cells were electroporated using the Neon transfection system with 9 μg TLR7-encoding plasmid (pUNO1-mTLR07-HA3x, InvivoGen, San Diego, CA) and seeded onto glass coverslips in a 24-well plate. Electroporation without any plasmid was used as the TLR7^−^ control. After 48 hr, cells were transfected with 2.5 μg luciferase IVT mRNA/well using Lipofectamine 2000; 24 hr later, cells were fixed with 1% paraformaldehyde in PBS for 10 min, permeabilized with 0.2% Triton X-100 in PBS for 5 min, and then assayed for TLR7 and IRAK4 proximity.

### *In Vitro* PLAs on Cells with Differential RIG-I and MDA5 Expression

RAW-Lucia ISG mouse macrophage cells, which express both RIG-I and MDA5, were used as WT. RAW-Lucia ISG-KO-RIG-I cells and RAW-Lucia ISG-KO-MDA5 cells were used as KO controls. All RAW-Lucia ISG cell lines were purchased from InvivoGen and maintained in DMEM with 10% fetal bovine serum (FBS) (Hyclone, Waltham, MA) and 100 μg/mL Normocin (InvivoGen, San Diego, CA). In all cases, cells were seeded onto glass coverslips in a 24-well plate and transfected with 2.5 μg luciferase IVT mRNA per well using Lipofectamine 2000. Cells were fixed with ice-cold methanol at 24 hr following IVT mRNA transfection for 15 min at −20°C, permeabilized with 0.2% Tween-20 for 5 min, and then assayed for RIG-I and MDA5 proximity with MAVS. We found that methanol fixation, rather than paraformaldehyde fixation, was essential for performing the MDA5 and RIG-I PLAs (data not shown).

### *In Vitro* PLAs on Cells with Differential TLR4 Expression

RAW 264.7 macrophages (ATCC, Manassas, VA), a commonly used cell line for studying the immune response, were used. They were maintained in DMEM with 10% FBS and 1% penicillin streptomycin (PS) (Thermo Fisher Scientific, Waltham, MA). Cells were transfected with and without 100 nM TLR4 siRNA (Thermo Fisher Scientific, Waltham, MA) delivered with Lipofectamine 2000. Cells were plated in a 96-well glass-bottom plate. 24 hr after siRNA delivery, 1 μg/mL LPS was added to TLR4-TIRAP conditions and 10 μg/mL was added to TLR4-TRAM conditions. After 30 min, cells were fixed with 1% paraformaldehyde in PBS for 10 min, permeabilized with 0.2% Triton X-100 in PBS for 5 min, and then assayed for TLR4 proximity with TIRAP or TRAM.

### *In Vitro* PLA Procedure

After permeabilization, cells were blocked against nonspecific binding with 1% BSA, 2% donkey serum, and 0.2% gelatin in 1× PBS for 30 min at 37°C. Primary antibodies were then incubated with cells in 1% BSA, 0.2% donkey serum, and 0.2% gelatin in 1× PBS at the indicated concentrations for 30 min at 37°C. Cells were washed in wash buffer A (Sigma-Aldrich, St. Louis, MO) for 10 min at room temperature (RT) and incubated with secondary antibodies (Sigma-Aldrich, St. Louis, MO) at manufacturer-recommended concentrations in 0.2% Tween-20 in 1× PBS for 30 min at 37°C. Cells were again washed in wash buffer A for 10 min at RT and then incubated with the ligase and ligase buffer, as specified by the manufacturer (Sigma-Aldrich, St. Louis, MO) for 30 min at 37°C. Next, cells were washed in wash buffer A for 10 min at RT and incubated with the polymerase and rolling circle amplification buffer, as specified by the manufacturer (Sigma-Aldrich, St. Louis, MO) for 100 min at 37°C. Finally, cells were washed in wash buffer B (Sigma-Aldrich, St. Louis, MO) for 20 min at RT and then 0.01× buffer B for 4 min at RT. Mounting medium with DAPI (Sigma-Aldrich, St. Louis, MO) was added and cells were imaged.

### Fluorescence Imaging

*In vitro* images were taken using a Hamamatsu Flash 4.0 version (v.)2 sCMOS camera on a PerkinElmer UltraView spinning-disk confocal microscope on a Zeiss Axiovert 200 M body. A 63×, numerical aperture (NA) 1.4 Zeiss Plan-Apochromat oil objective was used for all images. Imaging was controlled by Volocity acquisition software (PerkinElmer, Waltham, MA). Image stacks were recorded at 200-nm intervals.

### *In Vitro* PLA Quantification and Statistics

After images were acquired, Volocity acquisition software was used to draw regions of interest (ROIs) around each cell. Volocity software then was used to quantify the number of fluorescent puncta in each ROI above a specified signal threshold. Thirty cells were counted for each experimental condition. Two different statistics methods were used, depending on the number of time points in the experiment. For single-time point experiments, a one-way Kruskal-Wallis ANOVA test was used with a Dunn’s multiple comparisons test. For multiple-time point experiments, a two-way ANOVA was used with a Tukey’s multiple comparisons test. Statistics were performed in GraphPad Prism 7.0.

### *In Vitro* PLA Time Courses for Soluble Agonists

RAW 264.7 macrophages were plated in a 96-well glass-bottom plate. Soluble agonists included 8 μg/mL imiquimod (Sigma-Aldrich,St. Louis, MO) for TLR7, 10 μg/mL 5′ppp dsRNA and LyoVec (InvivoGen, San Diego, CA) for RIG-I, 0.1 μg/mL poly(I:C) and LyoVec (InvivoGen, San Diego, CA) for MDA5 agonist, 1 μg/mL LPS (Sigma-Aldrich, St. Louis, MO) for the TLR4-TIRAP pathway, and 10 μg/mL LPS (Sigma-Aldrich, St. Louis, MO) for the TLR4-TRAM pathway. Cells were fixed at 30 min and 6 hr after delivery with 1% paraformaldehyde for 10 min for TLR7 and TLR4 or ice-cold methanol at −20°C for 20 min for RIG-I and MDA5. Also at 6 hr, agonists were removed and fresh media were added to cells for 3 hr before fixing with paraformaldehyde or methanol. Cells were then permeablized as before and assayed for their respective interactions.

### Immunofluoresence Colocalization with PLA

A549 cells (ATCC, Manassas, VA) were incubated with CellLight Early Endosome-GFP, BacMAM 2.0 (Thermo Fisher Scientific, Waltham, MA) at 50 particles per cell for 16 hr prior to stimulation. Cells were then stimulated as indicated above for TLR7 and TLR4 and fixed 30 min post-delivery with 1% paraformaldehyde for 10 min. Cells were then permeablized as before and assayed for their respective interactions. RAW 264.7 macrophages were incubated with 200 nM MitoTracker Deep Red FM (Thermo Fisher Scientific, Waltham, MA) for 15 min and then stimulated with 0.1 μg/mL poly(I:C) and LyoVec for 30 min before fixation with ice-cold methanol at −20°C for 20 min. Cells were then permeablized as before and assayed for their RIG-I or MDA5 interactions.

### TNF-α ELISA

RAW 264.7 macrophages were plated in a 96-well glass-bottom plate. Soluble agonists included the following: 8 μg/mL imiquimod (Sigma-Aldrich, St. Louis, MO), 10 μg/mL 5′ppp dsRNA and LyoVec (InvivoGen, San Diego, CA), 0.1 μg/mL poly(I:C) and LyoVec (InvivoGen, San Diego, CA), and 1 and 10 μg/mL LPS (Sigma-Aldrich, St. Louis, MO). Supernatants were collected at 30 min and 6 and 24 hr post-stimulation before fixation, as previously described. A TNF-α ELISA (R&D Systems, Minneapolis, MN) on the supernatants was then performed according to the manufacturer’s instructions.

### IVT mRNA

IVT mRNA encoding firefly luciferase was provided by CureVac AG (Tübingen, Germany). It included a 5′ cap, the 3′ UTR from human albumin, as well as a poly(A) tail. All GFP mRNA contained identical sequences, a 5′ cap, the 3′ UTR sequence from mouse alpha globin, and a poly(A) tail. If modified, IVT mRNAs were made with 5-methylcytosine and pseudouridine.

### *In Vitro* PLA Time Courses for IVT mRNA

RAW 264.7 macrophages were plated in a 96-well glass-bottom plate. 200 ng per well of modified IVT luciferase mRNA, modified IVT GFP mRNA, or unmodified IVT GFP mRNA was delivered with Lipofectamine 2000. Cells were fixed, permeablized, and assayed for interactions at 30 min or 6 or 24 hr after delivery, as previously detailed.

### MTRIP Labeling of IVT mRNA

Luciferase IVT mRNA for *in vivo* experiments was labeled with MTRIPs for the detection by histology. A cartoon showing MTRIP assembly is shown in Figure S3A, and more detailed procedures for synthesis of MTRIPs and IVT mRNA labeling are described in previous work.^30^, ^53^ Briefly, 2′O-methyl DNA chimeras, complementary to three different regions of the 3′ UTR of the luciferase IVT mRNA or four different sequences of the 3′ UTR of the GFP IVT mRNA, were purchased from Biosearch Technologies (Petaluma, CA) (Table 1). Each oligo also contained a 5′ biotin. First, free amine groups were labeled with either Cy3B N-hydroxysuccinimide (NHS) ester (GE Healthcare, Chicago, IL) or DyLight 680 NHS ester (Pierce, Waltham, MA), according to the manufacturers’ protocols, and unbound dye was removed by centrifugal filtration (3 kDa molecular weight cut-off [MWCO], Millipore, St. Louis, MO). To assemble MTRIPs, each fluorescently labeled biotinylated oligo was incubated with Neutravidin at a 5:1 molar ratio for 1 hr at RT. Unbound oligos were then removed by centrifugal filtration (30 kDa MWCO, Millipore, St. Louis, MO). To label IVT mRNA with MTRIPs, IVT mRNA was first incubated at 70°C for 10 min and then immediately placed on ice to remove secondary structures. IVT mRNA was then incubated with each MTRIP at a 1:1 molar ratio overnight at 37°C. Unlabeled probes were removed by filtration (200 kDa MWCO, Cole-Parmer, Vernon Hills, IL), and IVT mRNA was buffer exchanged into Ringer’s lactate (RiLa). The mRNA concentration was determined by 260-nm absorbance using the Nanodrop 2000 (Thermo Scientific, Waltham, MA).

**Table 1:** List of Oligonucleotide Sequences

Target	Sequence
Luciferase probe 1	5′-TTTT*TTT**UA**T*UCUCAT*GGUAGGCT*GA-3′
Luciferase probe 2	5′-TTTT*TTT**GA**T*GAAUAAGCT*AUUGAT*-3′
Luciferase probe 3	5′-TTTT*TTT**ACAGGG**T*GUUGGCT*UUACA-3′
GFP probe 1	5′-T*TTTTTT*GCAAGCCCC-GCAGAAGGT*-3′
GFP probe 2	5′-T*TTATTT*AGAGAAGAAGGGCAT*GG-3′
GFP probe 3	5′-T*TTTTT*ACCAAGAGG-T*ACAGGT*GC-3′
GFP probe 4	5′-T*TTTTTT**C**T*ACUCAGGCT*UUAUT*C-3′

Boldface type indicates 2′-O-methyl RNA linkage, and T* indicates a thymine base with a C6-amino modification.

### Preparation of CholK

CholK was prepared in five steps starting from Kanamycin A, according to the schematic described in Figure S3C and the procedures described below, which are adapted from Sainlos et al.^54^

#### Kanamycin-Cbz-Teoc_3_ 3

Kanamycin A (1.15 g, 2.38 mmol, 1 equivalent [eq.]) was dissolved in water (10 mL). Et_3_N (0.33 mL, 2.38 mmol, 1 eq.) and a solution of *N*-benzyloxycarbonyloxy-5-norbornene-2, 3-dicarboximide (744 mg, 2.38 mmol, 1 eq.) in dimethylformamide (DMF) (15 mL) were successively added, and the mixture was stirred at RT for 24 hr. A solution of 2-(trimethylsilyl)ethyl-4-nitrophenylcarbonate (2.30 g, 9.76 mmol, 4.1 eq.) in DMF (20 mL) and more Et_3_N (1.36 mL, 9.76 mmol, 4.1 eq.) were then added, and the yellow solution was stirred at 55°C for 48 hr. The mixture was then concentrated and the residue taken up with EtOAc, before washing with NaOH 1M (3 times), water, and brine. The organic fraction was dried over MgSO_4_ and concentrated *in vacuo*. The residue was purified by flash chromatography (dethylene dichloride [DCM]/methanol [MeOH]) to afford the expected product (1.22 g, 49%). ^1^H nuclear magnetic resonance (NMR) (MeOD, 25°C) results were as follows: δ 0.02–0.05 (m, 27H), 0.98–1.02 (m, 6H), 1.50–1.53 (m, 1H), 2.04–2.08 (m, 1H), 3.19 (t, J = 9.4 Hz, 1H), 3.41–3.75 (m, 14H), 4.11–4.16 (m, 7H), 5.03–5.10 (m, 4H), 7.29–7.38 (m, 5H); mass spectrometry (MS) (ESI+) 1,074.5 [M+Na]^+^.

#### Kanamycin-Teoc_3_ 4

To a solution of *Kanamycin-Cbz-Teoc*_*3*_ (1.3 g, 1.23 mmol, 1 eq.) in degassed wet MeOH (containing 10% H_2_O, 50 mL) was added 10% Pd/C (0.17 g, 0.1 eq.). 3 cycles of vacuum-N_2_ were applied, followed by 3 cycles of vacuum-H_2_. The mixture was stirred overnight, then filtered over a pad of cellite and washed with EtOH. The filtrate was concentrated *in vacuo* and the residue purified by flash chromatography (DCM-MeOH) to afford the expected free amine (0.671 g, 59%). ^1^H NMR (MeOD, 25°C) results were as follows: δ 0.00–0.02 (m, 27H), 0.93–0.95 (m, 6H), 1.46–1.52 (m, 1H), 1.98–2.07 (m, 1H), 2.59–2.64 (m, 1H), 2.92–2.96 (m, 1H), 3.04–3.09 (t, J = 9.3 Hz, 1H), 3.32–3.57 (m, 4H), 3.60–3.69 (m, 9H), 4.03–4.13 (m, 7H), 5.01 (s, 2H), 5.12 (s, 1H); MS (ESI+) 939.6 [M+Na]^+^.

#### (Cholesteryloxy-3-carbonyl)]Kanamycin-Teoc_3_ 5

Et_3_N (0.12 mL, 1.5 mL, 0.87 mmol, 1.2 eq.) and cholesteryl chloroformate (0.330 g, 0.73 mmol, 1 eq.) were added to a solution of *Kanamycin-Teoc*_*3*_ (0.670 g, 0.73 mmol, 1 eq.) in tetrahydrofuran (THF; 70 mL) and DMF (15 mL). The mixture was stirred at RT overnight, then concentrated *in vacuo*. The residue was purified by flash chromatography (DCM-MeOH) to afford the expected product (0.655 g, 67%). ^1^H NMR (MeOD, 25°C) results were as follows: δ 0.00–0.02 (m, 27H), 0.67 (s, 3H), 0.82–1.59 (m, 39H), 1.84–2.02 (m, 6H), 2.27–2.29 (m, 2H), 3.07–3.09 (m, 1H), 3.33–3.39 (m, 6H), 3.53–3.69 (m, 9H), 3.86–3.89 (m, 1H), 4.02–4.11 (m, 6H), 4.55 (s, 1H), 5.01–5.01 (m, 2H), 5.34 (s, 1H); MS (ESI+) 1,352.0 [M+Na]^+^.

#### CholK

To a suspension of *(Cholesteryloxy-3-carbonyl)]Kanamycin-Teoc*_*3*_ (0.650 g, 0.49 mmol) in DCM (6 mL) was slowly added trifluoroacetic acid (TFA; 12 mL) at 0°C. The solution was stirred at RT for 2 hr and then concentrated. After 3 consecutive additions of MeOH and subsequent evaporations, the residue was purified by flash chromatography (reverse phase, C18-Silica, H_2_O + 0.05% TFA-MeOH) and lyophilized to give CholK (0.370 g, 61%). ^1^H NMR (MeOD, 25°C) results were as follows: δ 0.72 (s, 3H), 0.87–1.63 (m, 34H), 1.85–2.07 (m, 6H), 2.32–2.34 (m, 2H), 2.45–2.55 (m, 1H), 3.17–3.19 (m, 1H), 3.35–3.88 (m, 15H), 4.30–4.50 (m, 1H), 5.10 (s, 1H), 5.26 (s, 1H), 5.38 (s, 1H); MS (ESI+) 919.4 [M+Na]^+^.

#### Preparation and Sizing of CholK-mRNA Complexes

CholK was prepared by In-Cell-Art (Nantes, France). To prepare RNA-CholK nanoparticles, IVT mRNA and CholK were individually diluted in RiLa to 20 μL and mixed to generate a low charge ratio (+/−) particle (10 μg mRNA and a 1:10 charge ratio [+/−] of CholK:mRNA). The CholK solution was then added to IVT mRNA, mixed well, and allowed to incubate for 15 min prior to injection. Size and zeta potential of particles were measured with the Malvern Instruments Zetasizer Nano ZS. Particles were diluted in PBS to measure size or deionized water to measure zeta potential.

#### i.m. Injection

Female BALB/c mice (Charles River Laboratories, Wilmington, MA) were anesthetized with 2.5% isoflurane. The region around their anterior tibialis was shaved and then swabbed with isopropanol. 10 μg (40 μL) IVT mRNA diluted in RiLa was then injected into the anterior tibialis using a 29G needle. The alternate leg served as a sham injection control. Mice were housed and manipulated under specific-pathogen-free conditions in the animal care facilities of Georgia Institute of Technology. All experiments were in accordance with the Institutional Animal Care and Use Committee.

### Tissue PLAs

For histological PLAs, samples were initially deparaffinized. Antigen retrieval and permeabilization were performed prior to PLA as described above. To perform PLA, samples were blocked for 1 hr with a solution containing 0.5% Tween-20, 0.1% Triton X-100, 0.1% gelatin (Aurion, Wageningen, the Netherlands), 2% donkey serum (VWR, Radnor, PA), and 1% BSA (EMD Biosciences, St. Louis, MO) in PBS. Samples were then stained with the indicated antibodies (anti-TLR7 and anti-IRAK4; anti-RIG-I and anti-MAVS; or anti-MDA5 and anti-MAVS) overnight at 4°C, diluted 1:500 in a solution of 0.25% gelatin, 0.5% donkey serum, and 1% BSA in PBS. All remaining steps were in accordance with the manufacturer’s instructions (Sigma-Aldrich, St. Louis, MO).

### Tissue PLA Imaging, Quantification, and Statistics

Images were acquired with an UltraVIEW spinning disk confocal microscope utilizing a Hamamatsu Flash 4.0 v.2 sCMOS camera. A 40×, NA 1.3 Zeiss EC Plan-Neofluar oil objective was used for all tiled images, and a 63×, NA 1.4 Zeiss Plan-Apochromat oil objective was used for all other images. Imaging was controlled by Volocity acquisition software (PerkinElmer, Waltham, MA). Image stacks were recorded at 350-nm intervals. Tiled images were stitched using Volocity. Six images were acquired of each tissue section, and a total of four animals were used per group per time point. The PLA signal-imaging frame was quantified using Volocity software. The PLA puncta were identified as objects with a signal above a minimum threshold. The sum of PLA signal volume was measured for each frame. Two different statistical methods were used, depending on the number of time points in the experiment. For single-time point experiments, a one-way Kruskal-Wallis ANOVA test was used with a Dunn’s multiple comparisons test. For multiple-time point experiments, a two-way ANOVA was used with a Tukey’s multiple comparisons test. Statistics were performed in GraphPad Prism 7.0.

### *In Vitro* PLAs on RAW Cells Transfected with IVT mRNA with and without MTRIP Label

RAW 264.7 macrophage cells were seeded onto 96-well plates. Cells were transfected with 250 ng/well modified or unmodified GFP IVT mRNA with and without MTRIP labels using Lipofectamine 2000. Cells were processed for PLAs 2 hr later, as described above.

### Tissue Immunohistochemistry

Extracted tissue was fixed overnight using 4% paraformaldehyde in PBS. Tissue was embedded in paraffin and sectioned to 5 μm thick. To stain tissue, sections were deparaffinized, antigen retrieval was performed in citrate buffer (Dako, Carpinteria, CA) for 20 min under steam, and then tissue was permeabilized in PBS with 0.1% Tween-20 (Calbiochem, Waltham, MA) (PBST) for 10 min. Tissue was blocked with 5% donkey serum in PBST, incubated with primary antibodies (at a 1:200 dilution) overnight at 4°C in PBST, washed three times in PBST, incubated with the secondary antibody for 1 hr in PBST, stained with DAPI, and then mounted with prolong gold (Life Technologies, Waltham, MA).

### Flow Cytometry and Statistics

Lymph nodes and muscle were removed with the assistance of the Fluobeam NIR imaging system (Fluoptics, Cambridge, MA), at the indicated time points. Muscle tissue was dissociated with Type IV collagenase (Worthington Biochemical, Lakewood, NJ) for 1.5 hr at 37°C with gentle agitation. Lymph node and muscle samples were then dissociated by straining through a 40-μm cell strainer with gentle agitation from a syringe plunger. Cells were washed in PBS + 2% FBS, blocked with Fc block (BD Biosciences, San Jose, CA) according to the manufacturer’s directions, and then stained with a panel of antibodies diluted 1:100 in PBS with Fc block for 30 min on ice. Cells were washed with PBS + 2% FBS, fixed with 4% paraformaldehyde in PBS for 10 min, washed with PBS + 2% FBS, and stored at 4°C until analysis. Flow cytometry was performed with a BD LSRII and analyzed with FlowJo software. Samples were stained using two different antibody panels, either anti-F4/80 BV421, anti-LYVE1 Alexa 488 (eBioscience, Waltham, MA), I-Ad PE, anti-CD11c APC-Cy7, and anti-CD11b Alexa 647 or anti-CD3 BV421, anti-CD45R Alexa 488 (BioLegend, San Diego, CA), anti-CD335 PE, anti-CD11b Alexa 647, and anti-CD19 APC-Cy7. IVT mRNA was labeled with DyLight 680. All antibodies were purchased from BD Biosciences unless otherwise noted. ANOVA followed by Hsu’s multiple comparison with best post analysis was performed to determine significance using JMP Pro software (SAS, Cary, NC).

## Author Contributions

E.L.B., K.H.L., and P.J.S. planned the experiments and interpreted the data. E.L.B., K.H.L., S.M.B., and D.V. performed experiments. P.B. provided RNA and B.P. provided delivery reagents. E.L.B., K.H.L., C.Z., and P.J.S wrote and edited the paper.

## Conflicts of Interest

P.B. is an employee of CureVac, which commercializes RNA-based vaccines. B.P. is an employee of In-Cell-Art, which commercializes lipidic aminoglycoside derivatives.

## Supplemental Information

Document S1. Figures S1–S10

Document S2. Article plus Supplemental Information
