# Supplementary material for: Proximity Ligation Assays for In Situ Detection of Innate Immune Activation: Focus on In Vitro-Transcribed mRNA
Source: Mol Ther Nucleic Acids. 2018 Nov 20;14:52–66. doi: 10.1016/j.omtn.2018.11.002 (PMC6304375; doi:10.1016/j.omtn.2018.11.002)
Supplement: Document S1. Figures S1–S10 [file mmc1.pdf]

OMTN, Volume *14*

Supplemental Information

Proximity Ligation Assays for *In Situ* Detection of Innate Immune Activation: Focus on *In Vitro*-Transcribed mRNA

Emmeline L. Blanchard, Kristin H. Loomis, Sushma M. Bhosle, Daryll Vanover, Patrick Baumhof, Bruno Pitard, Chiara Zurla, and Philip J. Santangelo

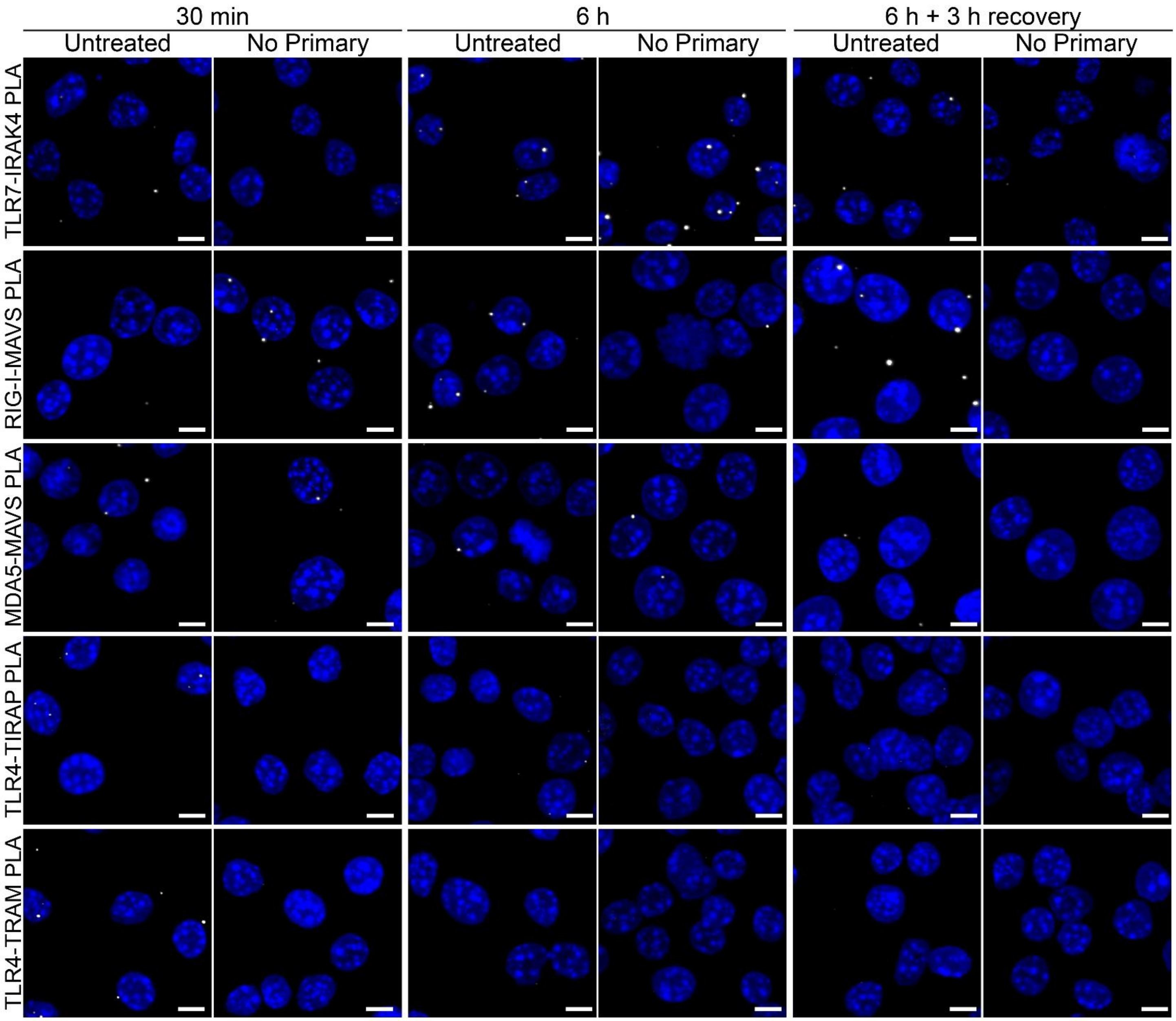

Figure S1: Representative images of controls for PLA of PRR activation kinetics. RAW 264.7 macrophages were incubated with the following agonists: 8 µg/ml of imiquimod for TLR7, 10 µg/ml of 5'ppp dsRNA for RIG-I, 0.1 µg/ml of poly(I:C) for MDA5, 1 µg/ml of LPS for TLR4-TIRAP, and 10 µg/ml of LPS for TLR4-TRAM or left untreated. Cells were fixed with 1% paraformaldehyde or methanol as previously indicated after 30 minutes or 6 hours. Also at 6 hours, agonists were removed and cells were incubated with media for 3 additional hours before fixing. PLA was performed between TLR7-IRAK4, RIG-I-MAVS, MDA5-MAVS, TLR4-TIRAP, and TLR4-TRAM. Representative images of PLA (white) are shown. Extended focus images are shown. Scale bar is 10 µm.

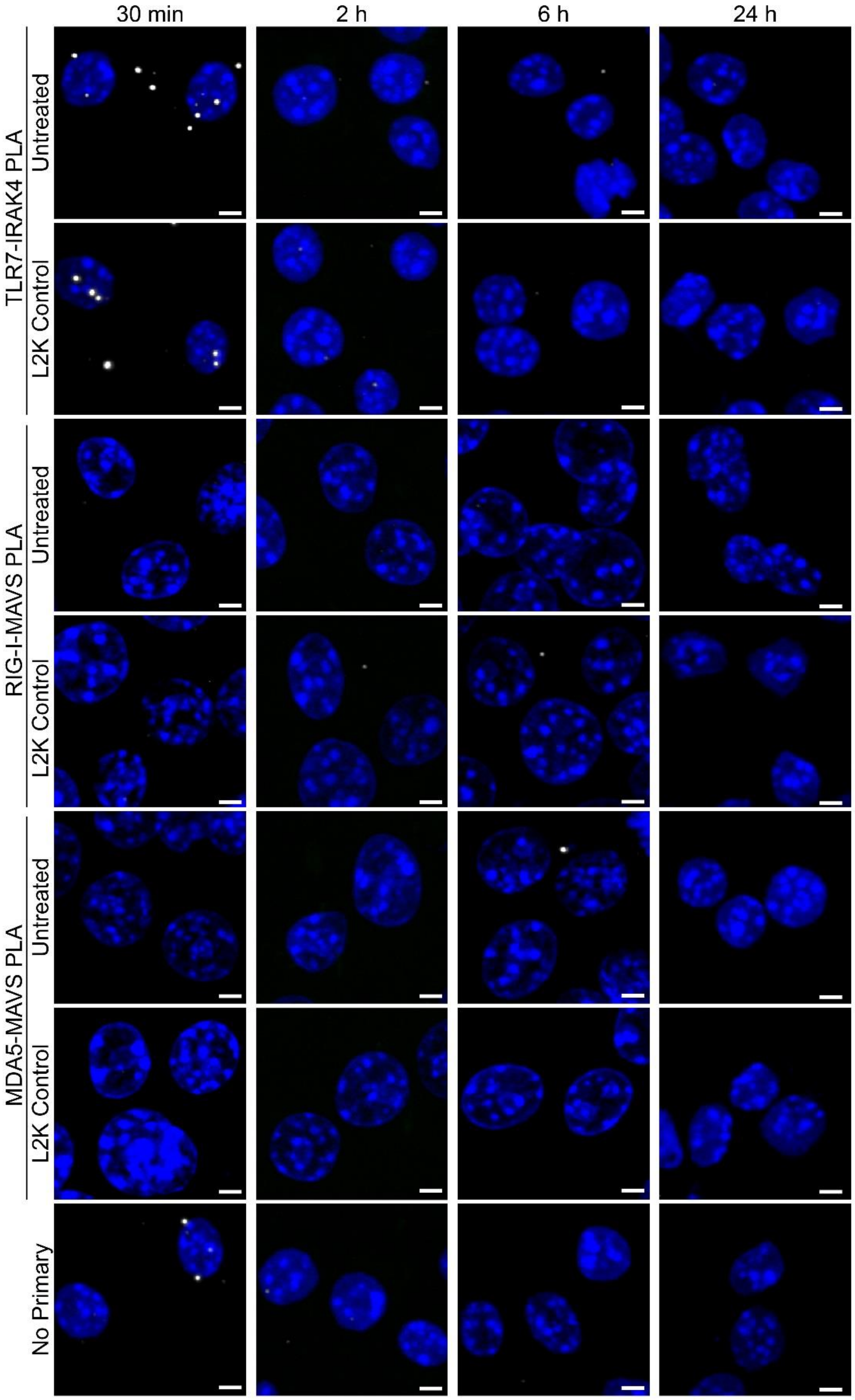

Figure S2: Representative images of controls for PLA of mRNA PRR activation. RAW 264.7 macrophages were either untreated, transfected with lipofectamine 2000 (L2K) alone, or transfected with 200 ng of unmodified or modified IVT GFP mRNA or 200 ng of luciferase IVT mRNA via L2K. Cells were fixed with either 1% paraformaldehyde or methanol after 30 minutes, 2 hours, 6 hours, or 24 hours. Representative images of PLA (white) are shown. Extended focus images are shown. Scale bar is 10 µm.

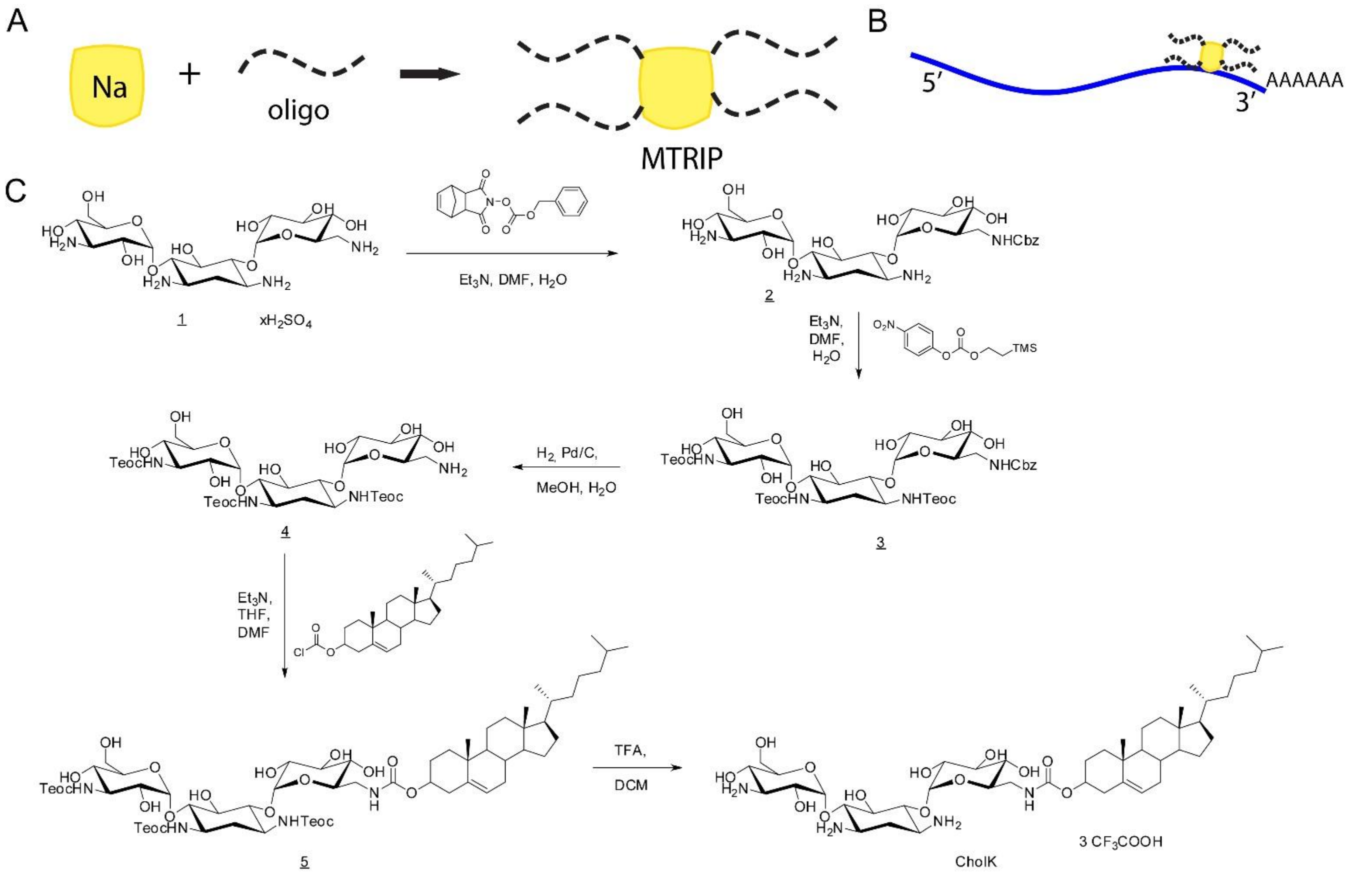

Figure S3: MTRIPs and CholK formulation. A) Schematic representation of MTRIP assembly (Na indicates Neutravidin). **B)** Schematic representation of mRNA labeling with a representative MTRIP. **C)** Details of CholK formulation.

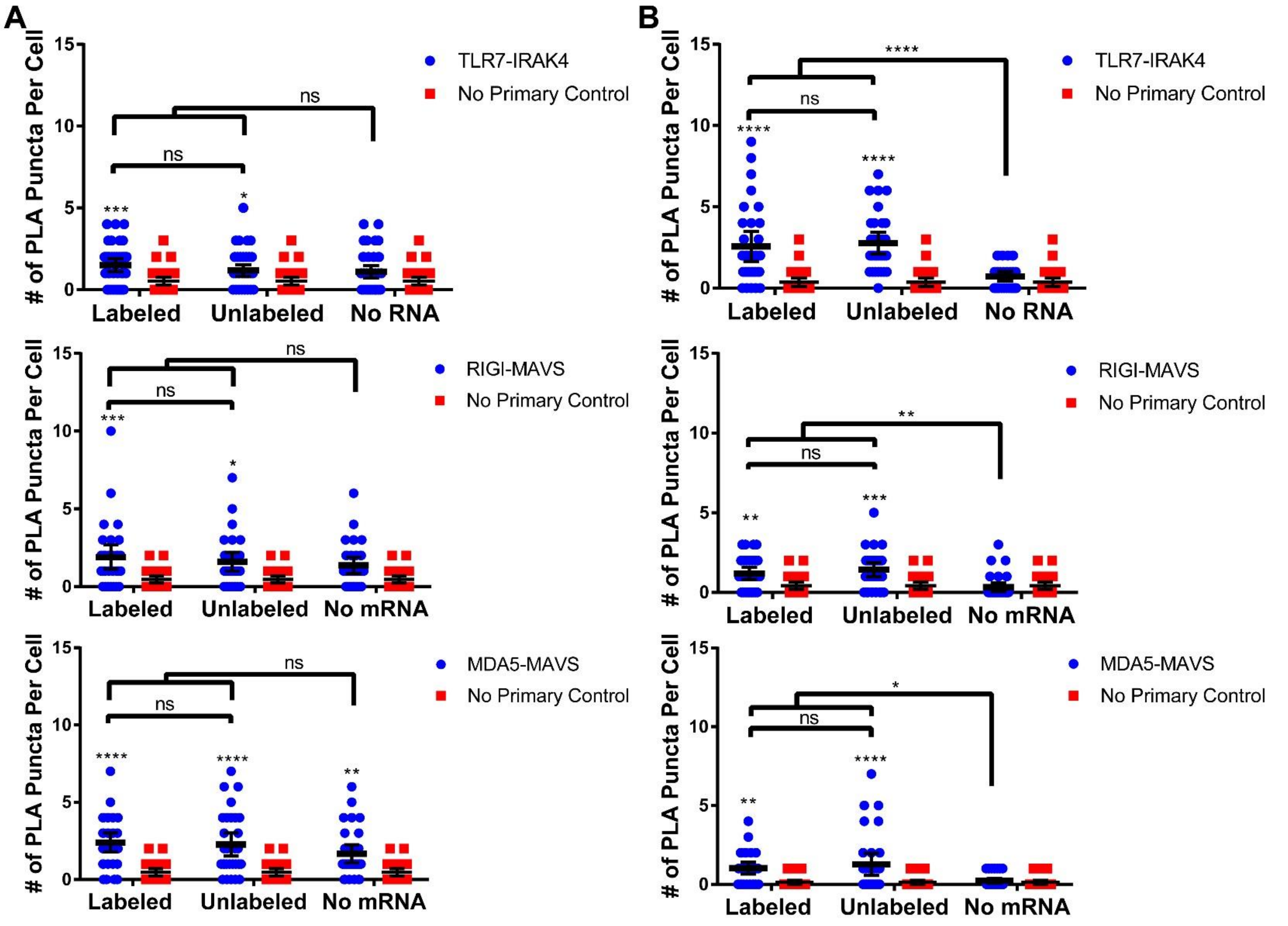

Figure S4: MTRIP labeling does not alter PRR activation of IVT mRNA. A) 250 ng of modified GFP mRNA were delivered to RAW 264.7 macrophages with Lipofectamine 2000. After 16 h, cells were fixed and PLA was performed. Statistics were performed with a two-way ANOVA with a Tukey's multiple comparison test, where n=30. * $p < 0.015$, ** $p < 0.0075$, *** $p < 0.005$ and **** $p < 0.0001$. 95% confidence intervals are shown in black. **B)** 250 ng of unmodified GFP mRNA were delivered to RAW 264.7 macrophages with Lipofectamine 2000. After 16 h, cells were fixed and PLA was performed. Statistics were performed with a two-way ANOVA with a Tukey's multiple comparison test, where n=30. * $p < 0.02$, ** $p < 0.07$, *** $p < 0.0002$ and **** $p < 0.0001$. 95% confidence intervals are shown in black.

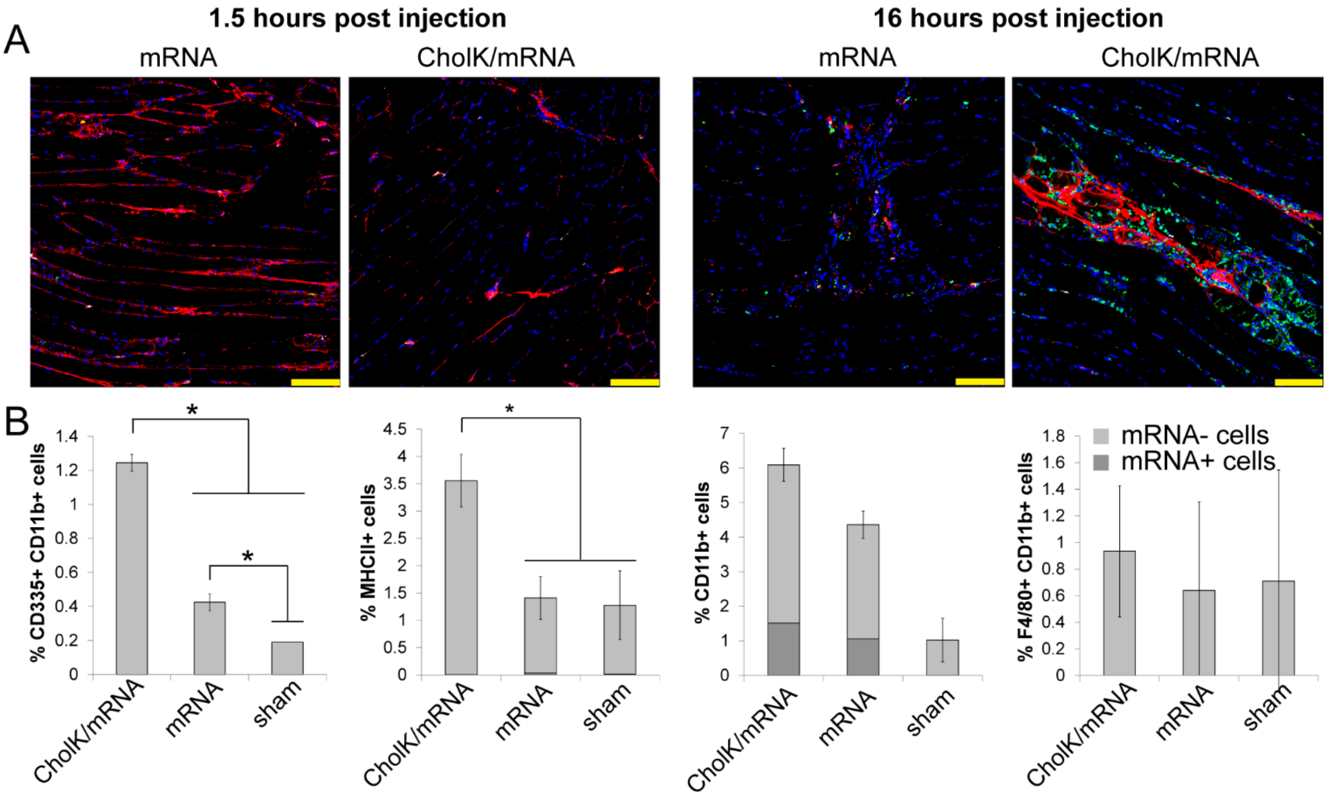

Figure S5: Distribution of IVT mRNA following injection and cellular uptake. A) Histological section of skeletal muscle tissue 1.5 and 16 hours following IM injection into the anterior tibialis of 10 µg of luciferase IVT mRNA formulated with CholK or unformulated. IVT mRNA is shown in red, CD11b staining is green, MHCII is white, and nuclei (DAPI) is blue. Scale bar is 100 µm. **B)** 16 hours following intramuscular injection, the whole anterior tibialis was dissociated into a single cell suspension and flow cytometry was performed to characterize the immune cells recruited to the muscle and the fraction containing IVT mRNA. Mean values and the standard error of measurement are shown (n=3). *indicates $p<0.05$, ANOVA followed by Hsu's multiple comparison with best.

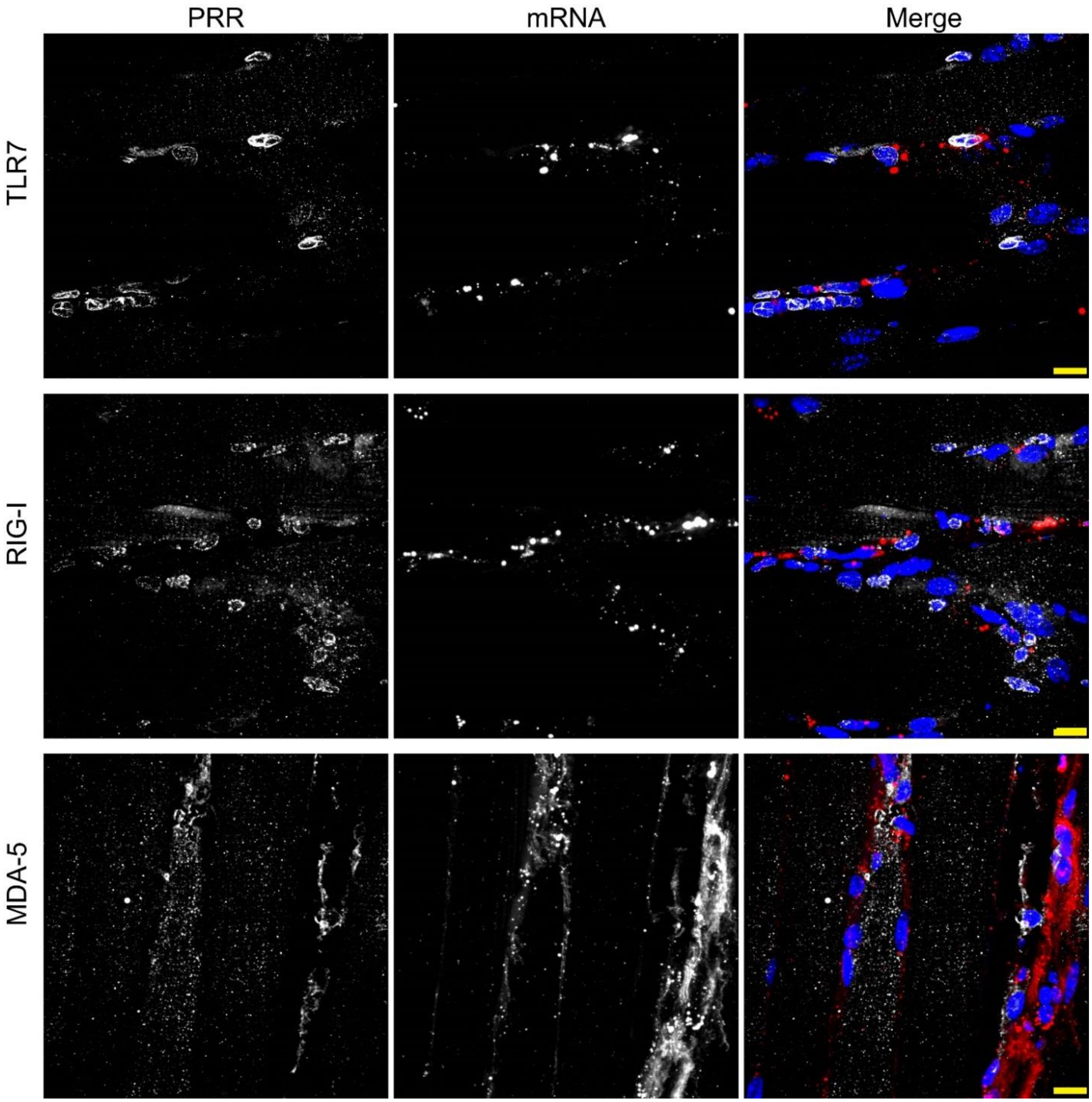

Figure S6: IVT mRNA uptake by TLR7+, RIG-I+, and MDA5+ cells. Histological sections of skeletal muscle tissue 16 hours following intramuscular injection into the anterior tibialis of 10 µg of MTRIP-labeled luciferase IVT mRNA (not formulated). In the merged image, the indicated PRR is shown in white, IVT mRNA is shown in red, and nuclei (DAPI) in blue**.** Scale bars are 12 µm.

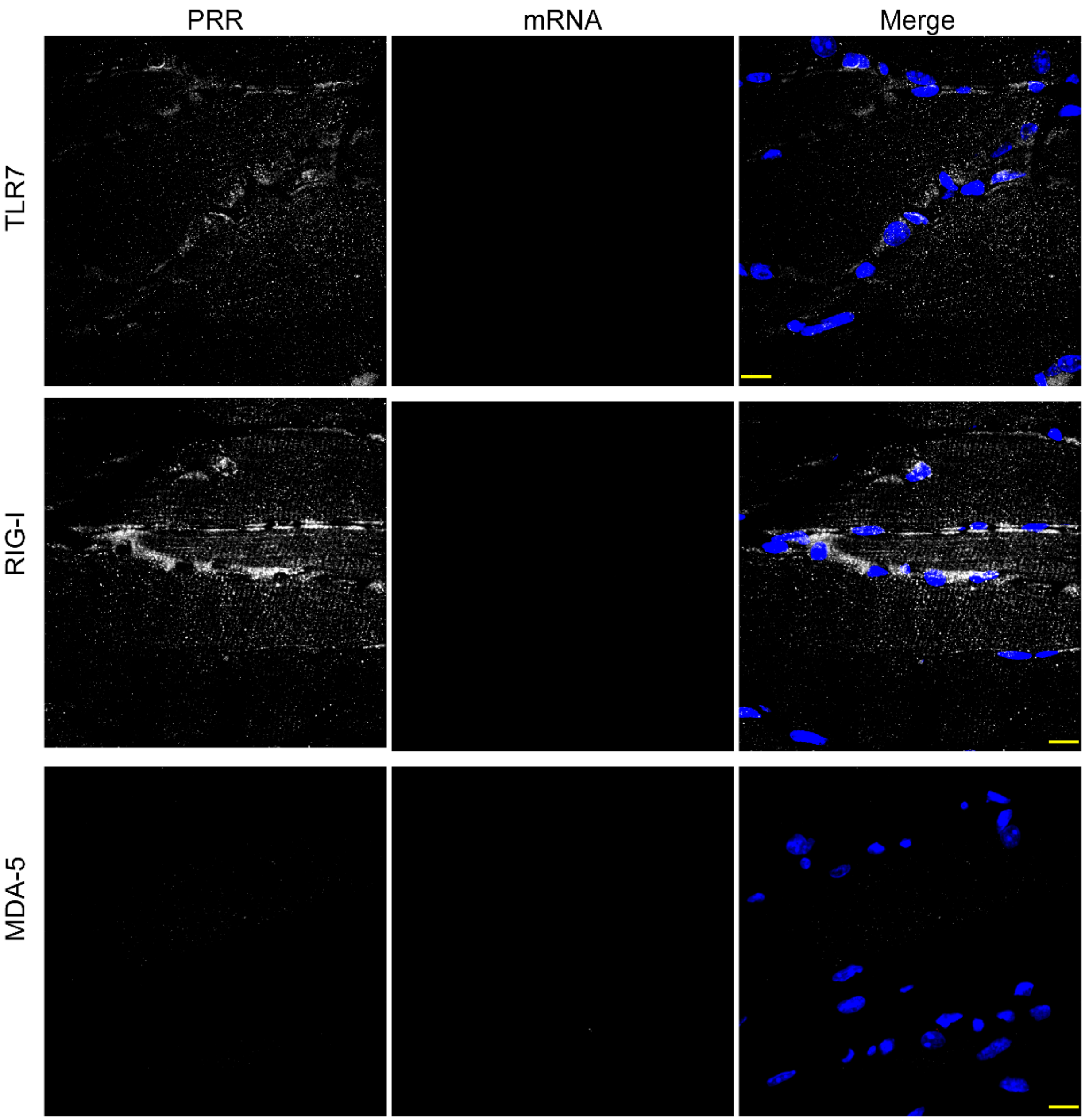

Figure S7: TLR7, RIG-I, and MDA5 detection in sham-injection skeletal muscle tissue. Histological sections of skeletal muscle tissue 16 hours following intramuscular injection into the anterior tibialis with RiLa. In the merged image, the indicated PRR is shown in white, and nuclei (DAPI) in blue. No signal is visible in the channel used for IVT mRNA imaging**.** Scale bars are 12 µm.

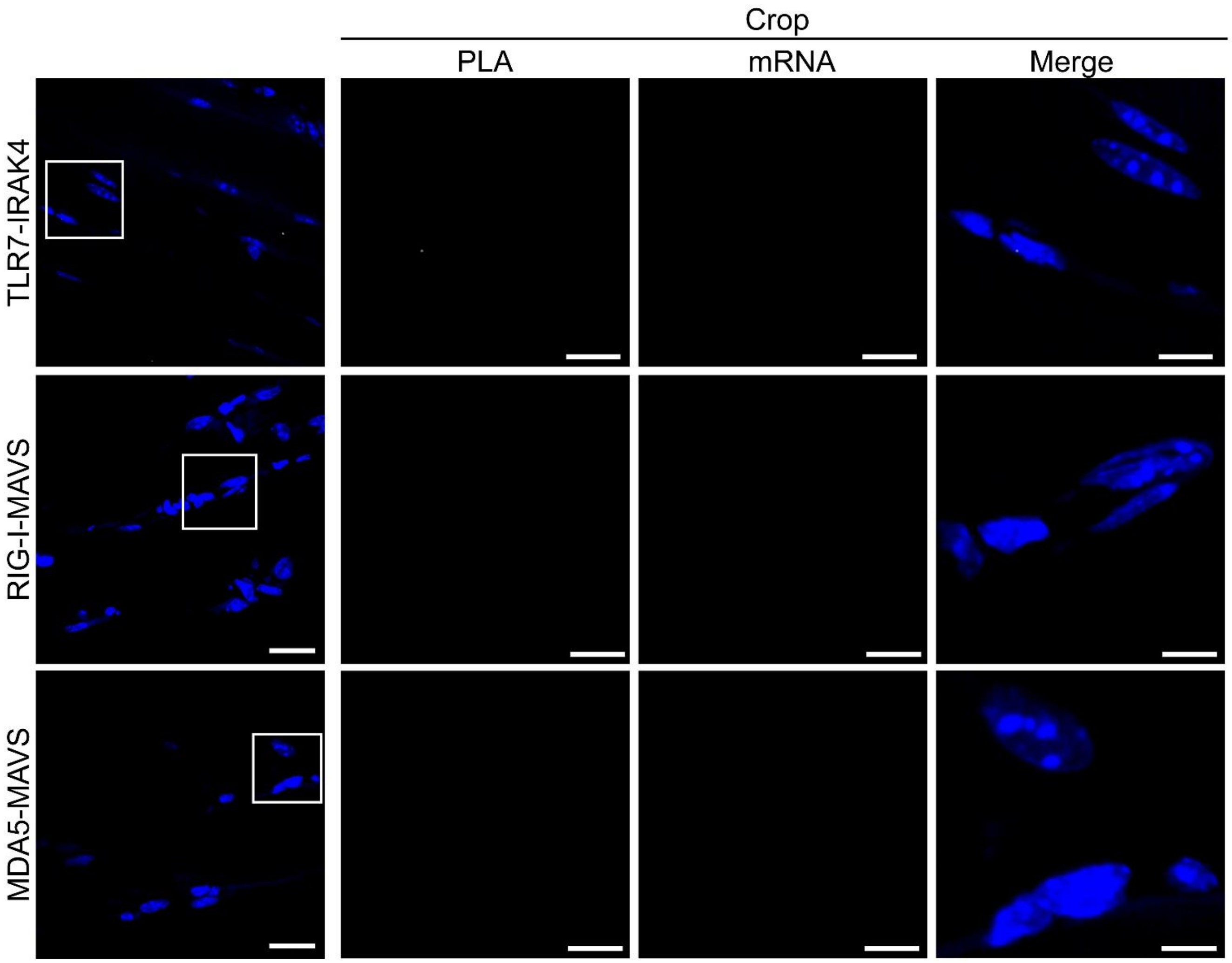

Figure S8: Representative images of PRR PLA following sham i.m. injection. Representative images of PRR PLA (white) 16 hours following IM injection into the anterior tibialis with RiLa. Extended focus images are shown. Scale bar is 20 µm for full images, 5 µm for crops. White boxes show cropped selection.

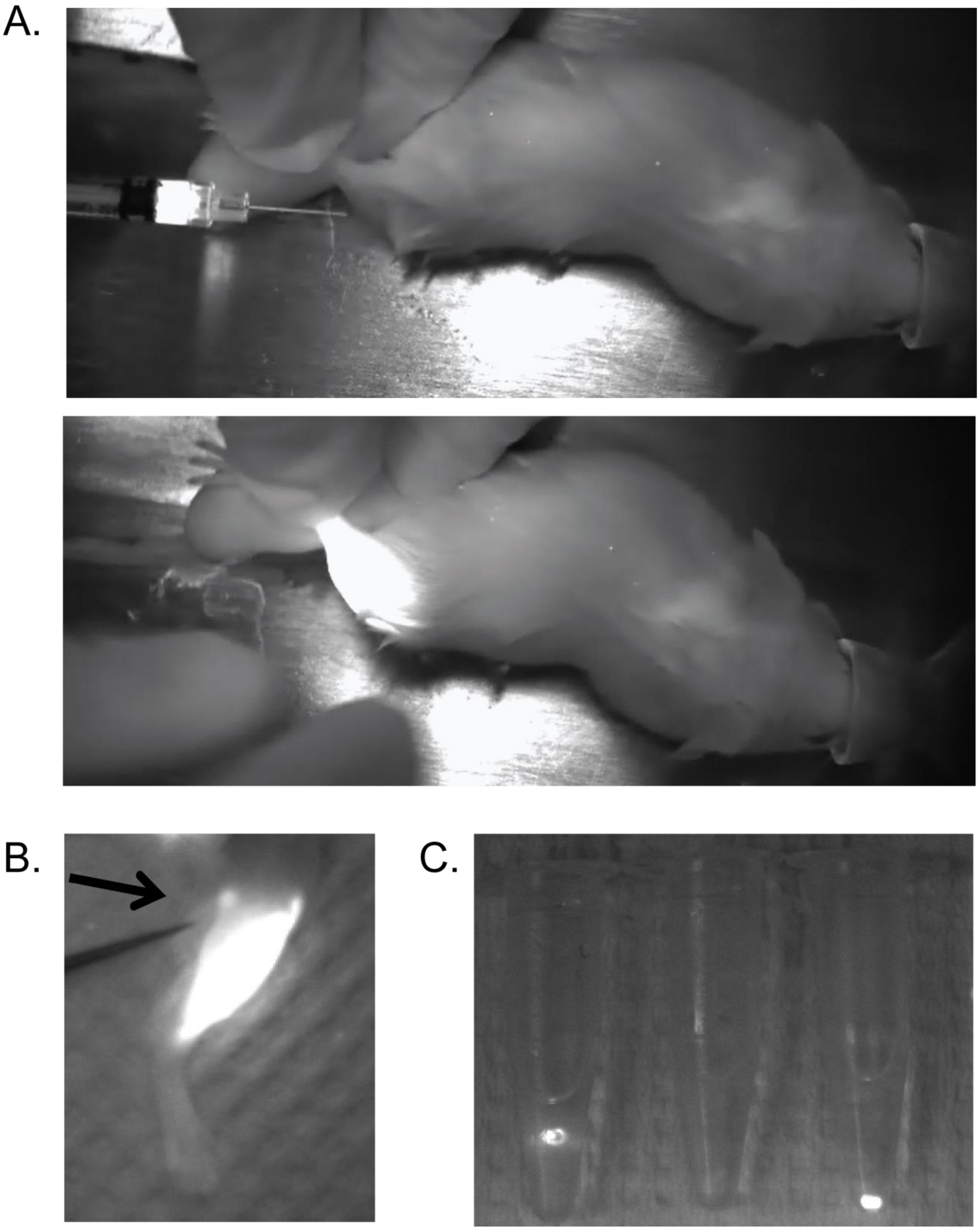

Figure S9: Representative images captured with Fluobeam of IVT mRNA following i.m. injection. A) Intramuscular injection of IVT mRNA into the anterior tibialis. **B)** Distribution of IVT mRNA following injection. The arrow identifies the popliteal lymph node. Image was acquired 16 hours following injection **C)** Lymph nodes collected 16 hours following IVT mRNA injection (from left to right: popliteal, subiliac, and lumbar aortic).

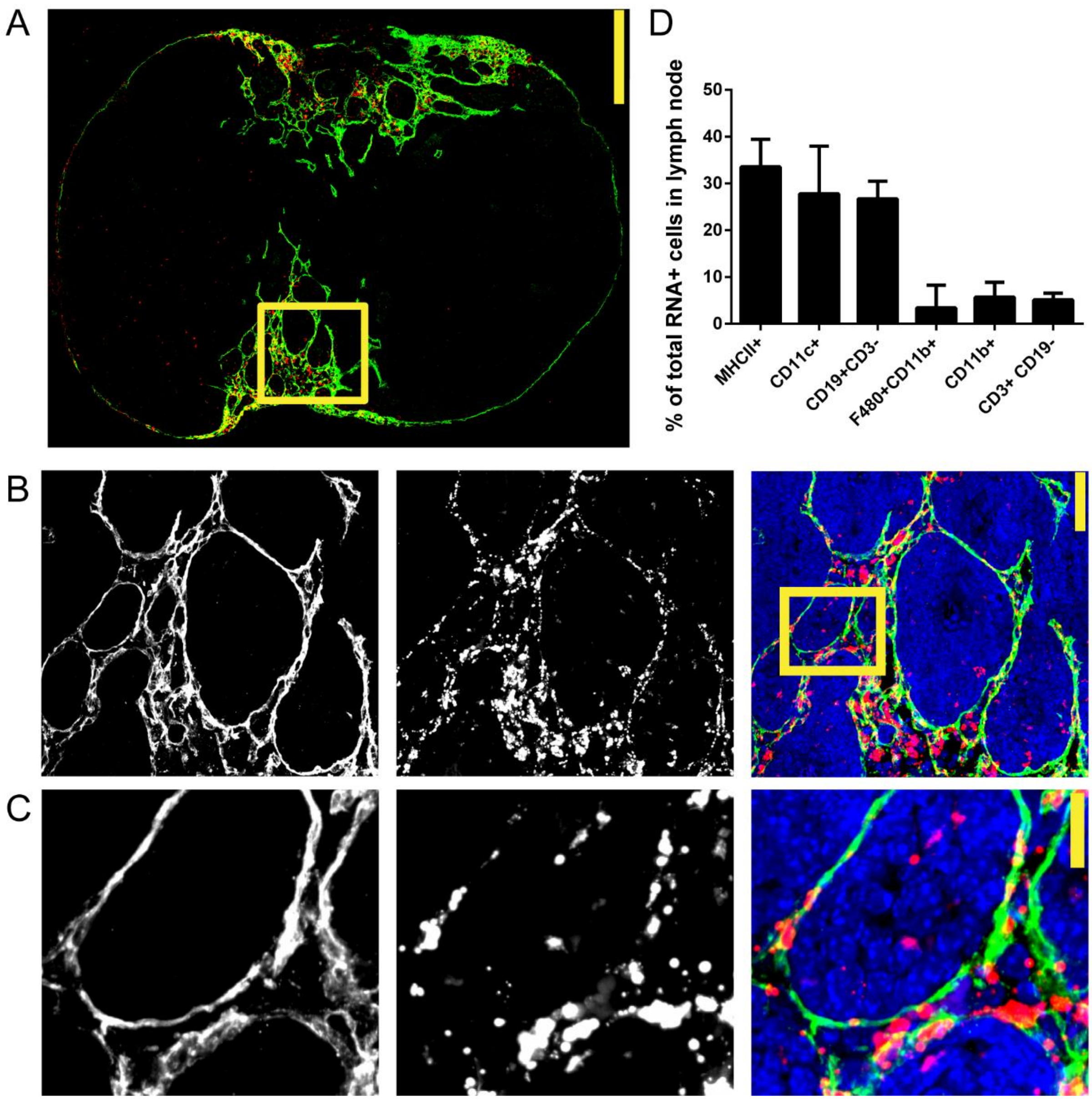

Figure S10: mRNA presence in lymph nodes 1.5 and 16 hours following injection. A) Histological cross-section of the entire lumbar aortic lymph node 16 hours following intramuscular injection into the anterior tibialis of 10 µg of luciferase IVT mRNA. IVT mRNA is shown in red, and lyve-1 staining is shown in green. IVT mRNA associates with lyve-1 staining, indicating presence in lymph node lymphatic vessels. Scale bar is 300 µm. **B)** Shows the blow-up image of the area indicated in (a) with scale bar of 50 µm and **C)** Shows the blow-up image of the area indicated in (b) with a scale bar of 18 µm. For both b) and c), the merged image shows mRNA in red, Lyve-1 in green and nuclei (DAPI) in blue. It is evident that mRNA is around lymphatic vessels, but does not always associate with lyve-1 positive cells. **D)** Mice were injected with MTRIP-labeled IVT mRNA formulated with CholK. 16 hours following injection, the lumbar aortic lymph node was removed and analyzed with flow cytometry. Lymph node cells were phenotyped with the indicated markers and the percent of cells containing mRNA for each cell type are shown for CholK/mRNA (n=3).
